# Assessment of open-field fluorescence guided surgery systems: implementing a standardized method for characterization and comparison

**DOI:** 10.1117/1.JBO.28.9.096007

**Published:** 2023-09-21

**Authors:** Marien I. Ochoa, Alberto Ruiz, Ethan LaRochelle, Matthew Reed, Eren Berber, George Poultsides, Brian W. Pogue

**Affiliations:** a University of Wisconsin Madison, Department of Medical Physics, Madison, Wisconsin, United States; b QUEL Imaging, White River Junction, Vermont, United States; c Cleveland Clinic - Marymount Hospital, Garfield Heights, Ohio, United States; d Stanford Medicine, Department of Surgery, Stanford, California, United States

**Keywords:** fluorescence-guided-surgery, standards, optics, imaging

## Abstract

**Significance:**

Fluorescence guided surgery (FGS) has demonstrated improvements in decision making and patient outcomes for a wide range of surgical procedures. Not only can FGS systems provide a higher level of structural perfusion accuracy in tissue reconstruction cases but they can also serve for real-time functional characterization. Multiple FGS devices have been Food and Drug administration (FDA) cleared for use in open and laparoscopic surgery. Despite the rapid growth of the field, there has been a lack standardization methods.

**Aim:**

This work overviews commonalities inherent to optical imaging methods that can be exploited to produce such a standardization procedure. Furthermore, a system evaluation pipeline is proposed and executed through the use of photo-stable indocyanine green fluorescence phantoms. Five different FDA-approved open-field FGS systems are used and evaluated with the proposed method.

**Approach:**

The proposed pipeline encompasses the following characterization: (1) imaging spatial resolution and sharpness, (2) sensitivity and linearity, (3) imaging depth into tissue, (4) imaging system DOF, (5) uniformity of illumination, (6) spatial distortion, (7) signal to background ratio, (8) excitation bands, and (9) illumination wavelength and power.

**Results:**

The results highlight how such a standardization approach can be successfully implemented for inter-system comparisons as well as how to better understand essential features within each FGS setup.

**Conclusions:**

Despite clinical use being the end goal, a robust yet simple standardization pipeline before clinical trials, such as the one presented herein, should benefit regulatory agencies, manufacturers, and end-users to better assess basic performance and improvements to be made in next generation FGS systems.

## 1 Introduction

Fluorescence guided surgery (FGS) has demonstrated improvements in surgical decision making and assessment of tissue function in real time for indicated procedures.^1^^,^^2^ Not only does fluorescence guidance provide a high level of structural perfusion accuracy in reconstruction cases, but it could also allow for molecular characterization of healthy versus diseased tissues in real time in future approved indications.^3^ The field evolved out of many decades of retinal flow imaging with indocyanine green (ICG) and started into bulk tissue perfusion imaging with 510(k) clearance of the SPY SP2000 FGS system through the United States Food and Drug administration (FDA) process.^4^ Since then, multiple FGS devices have been similarly cleared for use in open and laparoscopic surgery. The design of FGS devices is developed together with contrast agents, although advances in each are usually separated by their regulatory approvals. Despite the rapid growth^5^^–^^7^ in the use of fluorescence devices among surgeons and advances in contrast agents and FGS devices, there has been a lack of development of standardization methods within the field, and a recent consensus paper has called for improvement in this situation.^8^ This study examines these recommendations by implementing the suggested FGS assessment metrics for analysis of several of the leading commercial instruments.

For any growing field, standardization is a fundamental part of ensuring that advances in manufacturing and reliability, along with technology diversification, continue.^9^ The standardization process can also increase end-user understanding of the instrumentation, and especially for surgical devices, it can make their use safer in patient procedures, ensuring more consistency of use and in outcomes. The development of standards is also key in helping regulatory agencies, such as the FDA, in defining more robust pathways for decisions about the clearances of devices.^10^ Despite clinical use being the end goal, a robust yet simple standardization pipeline before clinical trials should benefit both regulatory agencies and manufacturers. Limitations in the creation of standards stem from the unique nature of FGS signals and are partly related to challenges in creating long term stable test targets that can assess the full range of pertinent system functionality and be suitable for the diverse range of systems existing today. Despite the range of commercial instruments, there are many commonalities inherent to optical imaging methods that can be exploited to produce such a standardization procedure. The first clear step in this pathway was developed by a solid distributable phantom and test target produced by Gorpas et al.^11^ Following this, a group of multiple researchers produced a consensus report^12^ suggesting the evaluation of FGS systems for the following criteria: (1) imaging spatial resolution and sharpness, (2) sensitivity and linearity, (3) imaging depth into tissue, (4) imaging system depth of field (DOF), (5) uniformity of illumination, (6) spatial distortion, (7) signal to background ratio, (8) excitation bands, and (9) illumination wavelength and power. These evaluation criteria would simultaneously facilitate FGS system performance assessment and inter-system comparisons, as well as multi-center studies, translation of clinical trial results into the standard-of-care, and ensuring patient outcomes are independent of the FGS system used.

This manuscript contains two main sections: (i) an overview of open-field imaging system components and parameters that can be quantified through commercially available photo-stable ICG phantoms^7^ and (ii) an assessment of five FDA-approved FGS systems using the proposed system characterization pipeline. Section 2 focuses on relevant aspects of open-field systems related to their clinical deployment, with an emphasis on ICG imaging and potential techniques to quantify the performance metrics suggested in the consensus report.^12^ In addition, Sec. 2 describes the implementation and results obtained from the proposed FGS system characterization pipeline for five FDA approved open-field FGS systems. Of importance, the use of *in vitro* solid phantoms avoids the uncertainty that might arise from *in vivo* tissue interactions. Hence this acts as a preliminary step to better assessing key features of FGS systems before clinical trials, in which tissue variations might be hard to control. The discussion section clarifies the choice of phantoms and compares the results for future development and further optimization of standard choices in the setting of FGS.

## 2 Overview of Open-Field FGS Contrasts, Components, and Parameters

### 2.1 Overview of Several Approved FDA Systems and Contrast Agents

**Fig. 1:**
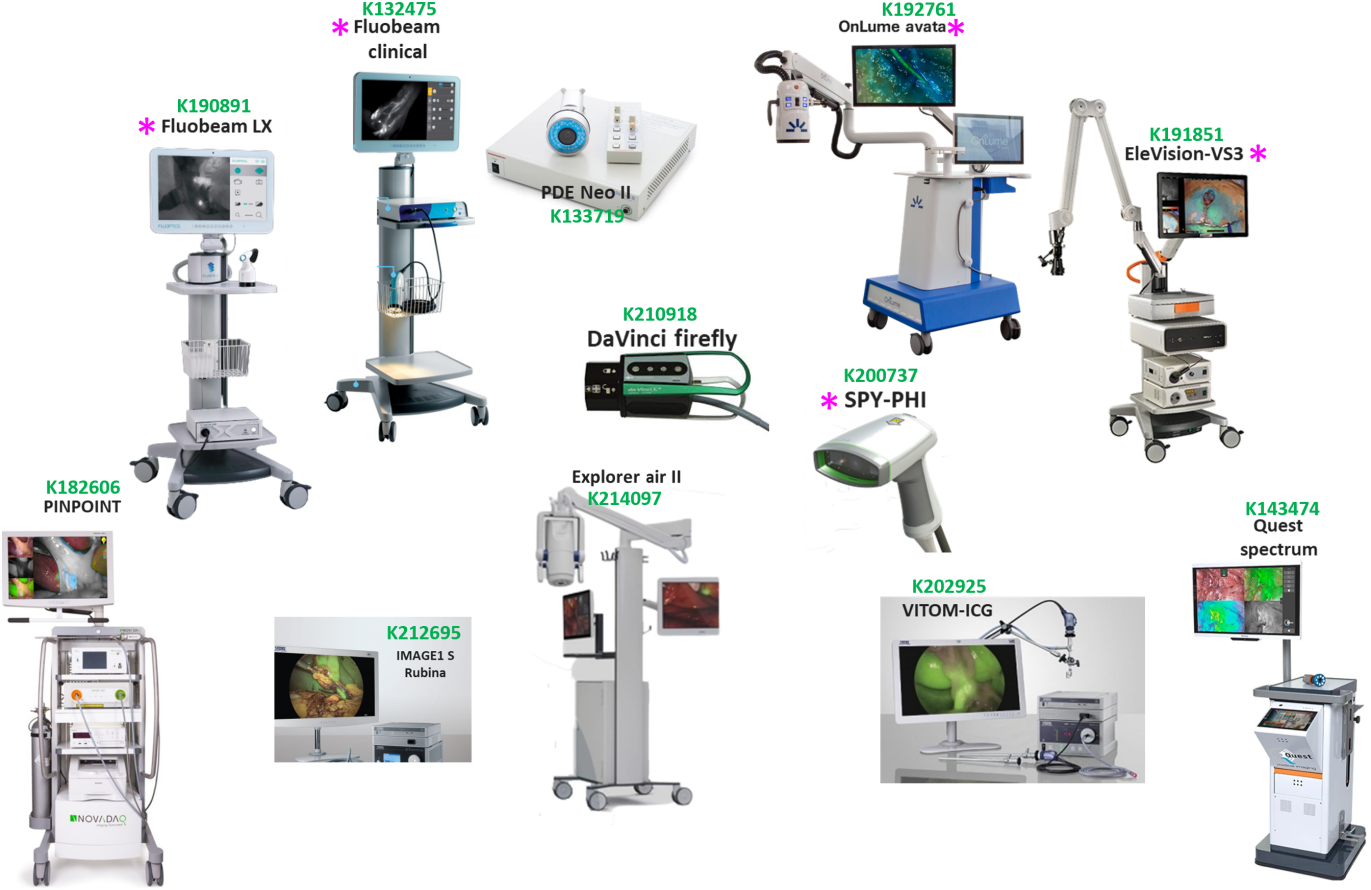
Examples of several FDA cleared systems. 510(k) clearance numbers are also provided for further understanding of approved indications for use. Systems displaying an asterisk (*) were used in this work for evaluation of the proposed standardization procedure.

Multiple FGS devices have been cleared for clinical use under the current FDA 510(k) pipeline. Figure 1 highlights examples of FDA cleared FGS devices, and 510(k) numbers are provided as a guide to the approved uses of each system. As described in the highlighted 510(k) summary of each device, common indications of FGS systems include visualization of blood-flow and related tissue perfusion, as well as related tissue-transfer in free flaps during micro and reconstruction surgeries. Nevertheless, clinical trials have explored numerous uses that might be particular to individual devices, some of them being encompassed within 510(k) indications of use. For example, in gallbladder removal surgery (laparoscopic cholecystectomy) the RUBINA™ guided surgery system combined with an ICG contrast agent has helped to identify biliary and vascular tissues, as well as the presence of abnormalities that can cause complications during surgery.^13^

The PINPOINT system has been used together with an ICG contrast agent for identification and resection of liver hepatoblastomas that otherwise would not be detected, hence improving the patient survival rate post-surgery.^14^ The use of DaVinci Firefly,^15^ in which robotic gastrectomy (lymphadenectomy) can be performed in conjunction with an ICG contrast agent, showed that lymph nodes were better localized through FGS than through the traditional surgical methods. FGS platforms have also aided in the visualization of intestinal perfusion. In a case of mesenteric ischemia, the Elevision system was used to estimate which bowel tissue to resect during surgery.^16^ Real time cystectomy has also been performed through fluorescence guidance by the SPY-PHI system.^17^ Another important application is breast cancer removal and reconstruction surgeries in which FGS systems such as OnLume Avata^18^ and VITOM-ICG^19^ have improved surgery outcomes. In the case of the OnLume Avata FGS system, adaptations on its design have allowed for surgery under room light conditions. The VITOM-ICG setup has also been utilized in neurosurgery^20^ for 3D exoscopic visualization of cranial and spinal tissues. Even though ICG is the only major contrast agent approved for use in these FGS devices, multiple evolving contrast agents are of future interest. Beyond ICG, there are several system developments aimed at other contrasts or endogenous fluorescence signals. Devices such as Fluobeam Clinical^21^ and Fluobeam LX^21^ have been used to image the near-infrared (NIR) autofluorescence of thyroid/parathyroid glands, which can be excited around 785 nm with emission peaks from 820 to 830 nm. Of importance, these emission and excitation profiles overlap with those of ICG. Another contrast agent explored is 5-ALA, which promotes intracellular generation of protoporphyrin IX (PpIX) in cancer cells.^22^ Imaging of PpIX has been widely deployed in neurosurgery with the approved formulation of Gleolan^23^ and in the bladder with the approved formulation Cysview.^24^ As described by the review works of Pogue et al.^25^ and Hadjipanayis,^23^^,^^26^ 5-ALA induced PPIX guided surgery is one of the great successes of FGS. Surgical microscopes such as the Leica FL400 (Leica, Illinois, United States) and Zeiss BLUE 400/OPMI Pentero 900^27^ have improved resection of abnormal brain tissues such as high grade gliomas, significantly changing patient outcomes. Even though this standardization work is limited to devices that allow for macroscopic open-field imaging rather than surgical microscopes due to differences in their optical arrangement, a similar standardization workflow could be implemented by accounting for the size of the microscopic field of view (FOV) in the used characterization phantoms. PPIX surgical microscopes (e.g., Leica and Zeiss) often have configurations for ICG imaging; hence with appropriate ICG phantoms that are scaled to their microscopic FOV, the proposed characterization procedure can be used. In addition to being limited to macroscopic open-field FGS systems, the current pipeline is also limited to systems that operate with ICG or where the contrast agent has overlapping excitation and emission profiles to those of ICG (e.g., parathyroid glands autofluorescence). This was due to the unavailability of photo-stable phantoms for contrasts different than ICG (e.g., PPIX). A similar characterization approach can be followed for FGS devices with contrast agents different than ICG in which photo-stability of the agent can be validated.

Methylene blue^28^ and fluorescein are also FDA approved agents. Pafolacianine, which is linked to an NIR dye with a peak excitation and emission at 776 and 796 nm, respectively, and contains ligands to folate receptor-alpha that are over-expressed in multiple types of cancer (e.g., ovarian and lung cancer), was cleared by the FDA in 2021.^29^ Examples of systems now aiming to be approved for pafolacianine detection are the EleVision IR, Quest Spectrum, and Explorer Air II systems.^30^ Other dyes that have been applied in clinical studies are Cy5, Cy7, IRDye800CW, SO456, IRDye 700DX, SGM-101, and ZW800-I.^31^ Despite all of this activity, ICG imaging accounts by far for the largest use of FGS in open surgery today and likely for the near future. Thus, throughout this standardization procedure analysis, ICG phantoms were used. Specially developed target NIR contrasts that are excited and emit at similar bands to ICG can also be characterized.^7^ Within all of the available FDA cleared systems, this work focuses on the analysis of the proposed standardization procedure for a subset of five FDA cleared devices: Fluobeam Clinical, Fluobeam LX, SPY-PHI, OnLume Avata, and EleVision systems.

### 2.2 Common Open-Field System Components and Quantitative Parameters

**Fig. 2:**
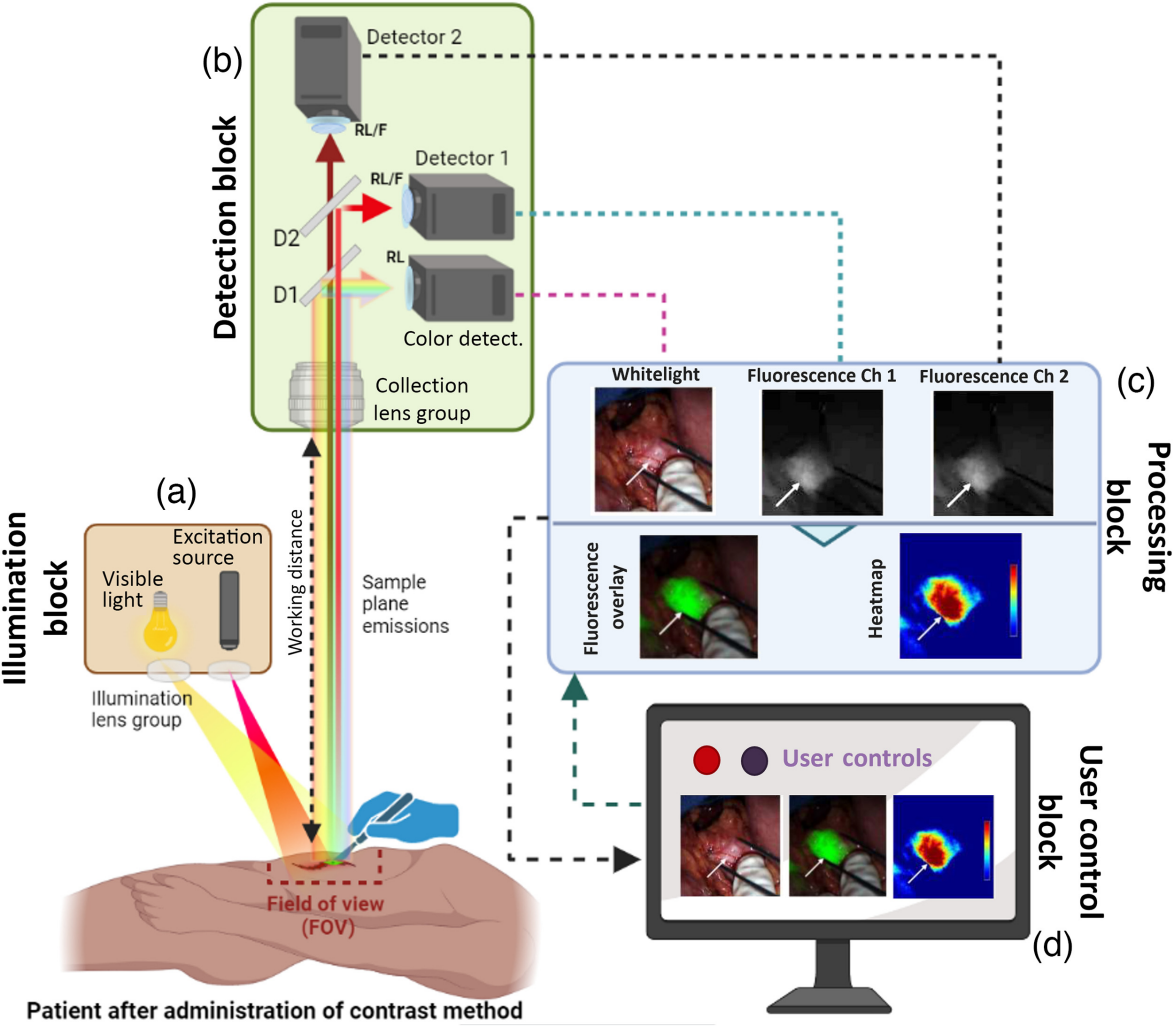
Main building blocks of a generic fluorescence guided surgery (FGS) system. (a) Illumination block containing excitation and visual aid illumination sources. (b) Detection block containing, here, three different detectors as an example, with two for fluorescence and one for white light images; relay lenses (RL); optical filters (F); and dichroic mirrors (D1 and D2).^5^^,^^32^ (c) Processing block in charge of image processing and rendering. Example figures displayed from Lu et al.^33^ with author permission. (d) Depiction of user interface for manipulation of image display options.

The selection of a contrast agent (exogenous or endogenous) is done with the development or choice of instrumentation that allows for its detection in the target tissue, even when the excited fluorescence is dimmer than the background intensity.^5^ A contrast agent can have a high quantum yield and high photo-stability, but the ability to detect its fluorescence and accurately resolve the pertinent fluorescent features is highly dependent on the FGS system components. In simplistic terms, an FGS system is composed of four main blocks, which are highlighted in Fig. 2 and are referred to throughout this manuscript as (i) illumination, (ii) detection, (iii) image processing, and (iv) user control blocks. One of the goals of the proposed standardization procedure is to quantify the performance of each of these blocks, except for the user control block in which only a description of useful features can be done. It is inevitable that most tests reveal a mixture of the influence of several of these system blocks; however, some are more definitive than others, as is highlighted below.

#### 2.2.1 Illumination block

**Fig. 3:**
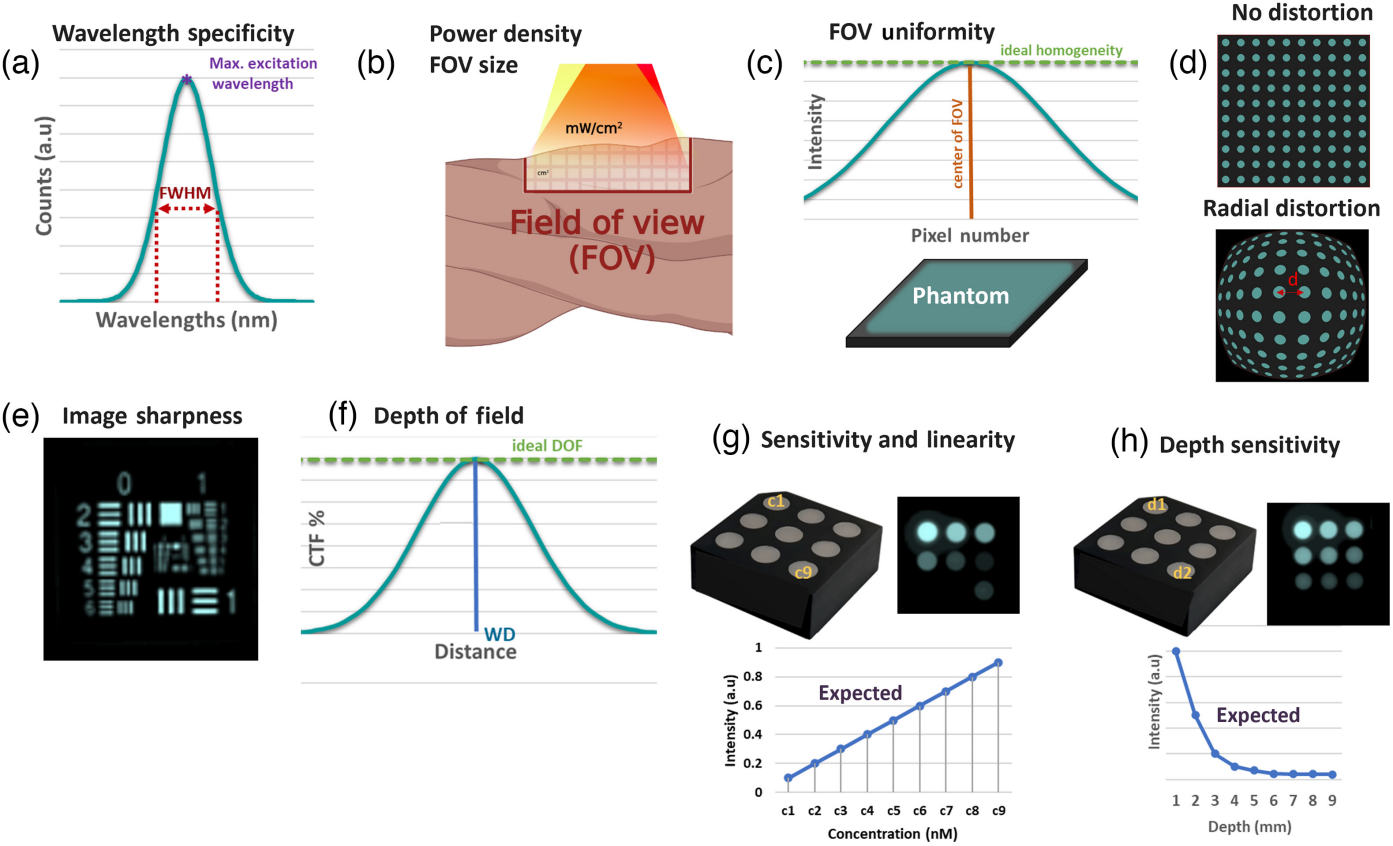
Summary of quantitative parameters tested with specific phantoms or measures. (a) Depiction of FWHM for an excitation band. (b) Graphic description of FOV and illumination power per unit area. (c) Graphic description of FOV uniformity and phantom used for characterizing it. (d) Comparison of systems with and without distortion and sample that can be used for characterization. (e) USAF 1951 fluorescent target that can be used for measuring resolution. (f) DOF depiction of fluorescent target and its CTF across different distances from the ideal focal plane or manufacturer defined WD. (g) Imaging phantom used for measurement of concentration sensitivity. (h) Imaging phantom used for measurement of depth sensitivity and expected trend.

The illumination block [Fig. 2(a)] is composed of an excitation source matching the absorption wavelength of the contrast agent, while avoiding the emission band to be detected and in many cases a white-light source, which is needed for the surgeon to properly see the surgical field. Important parameters of the excitation source are the optical radiance or flux density $\left[{\mathrm{mW}/\mathrm{cm}}^{2}\right]$, bandwidth [nm], homogeneity of illumination, and optical-tissue penetration depth. The excitation source power density must be high enough to excite the fluorophore without causing it to photo-bleach but low enough to not harm the patient. Due to safety and ease of use requirements, optical irradiance for FGS systems typically ranges from 10 to $25\;\mathrm{mW}/{\mathrm{cm}}^{2}$ at the FOV.^2^ Another important characteristic is the bandwidth of the excitation source. Typically with a Gaussian shape, as shown in Fig. 3(a), the excitation band is characterized by its full width half maximum (FWHM),^34^ in which a narrower peak and smaller FWHM are indicative of high wavelength specificity. Hence, a larger FWHM provides a broader spectral output, which might result in excitation of other endogenous components; although in most diode laser based systems, this spectral band is quite narrow. But the spectral band is of special concern for visible regime wavelengths in which autofluorophores such as FAD, NADH, ribovaflin, lipofuscin, or melanin are excitable. ^35^

Over the years, FGS systems have shifted into the NIR and NIR II regimes due to reduced autofluorescence bleed-through and scattering, hence avoiding major tissue autofluorophores and increasing imaging depth through tissue. There are cases in which contrast multiplexing is explored using a wider bandwidth, but most FGS systems aim at specific narrow excitation bands in the NIR to excite fluorescence of one contrast agent, with minimal leakage of this light into the detection band of the cameras. Laser diodes are the preferred method of excitation; however the use of light emitting diode (LED) sources has increased significantly, despite their broader spectral output, due to lower regulatory barriers in their use.^5^ Despite having to account for a loss in excitation power, LED based illumination blocks often add filters to provide higher specificity to the wavelength of interest. Spectrophotometer devices can collect the spectral distribution of the illumination source and FWHM values can be further retrieved. The use of pulsed illumination can also be implemented on this block. Pulsed illumination can then be combined with gated detection to temporally isolate signals from the ambient background. Some developing experimental systems use ultra-fast sampling to calculate nanosecond fluorescence lifetime values, which can be indicative of tissue environmental changes.^36^^,^^37^ Optical irradiance or flux density^38^ can be quantified through optical power meters that cover a mW/unit area range. In an ideal flat scenario in which an illumination source homogeneously covers the FOV, the optical power density is the same across unit area. Lenses and diffusers are often used to homogeneously distribute the excitation source in the FOV despite changes in source positioning. Knowing the illumination distribution of an FGS source with respect to the detector can help surgeons understand how the positioning of the instrument can account for topography differences in the patient FOV.

#### 2.2.2 Detection block

To image the fluorescence output within the FOV, the detection block [Fig. 2(b)] incorporates lenses, optical filters, and imaging detectors. In addition to the FOV uniformity, the effect of the FOV size is another important parameter to quantify. As depicted in Fig. 2(b), for open surgery systems, the emissions from the sample plane are collected by the detection block through a lens group. The lenses have to efficiently collect emissions from the established FOV at the determined working distance (WD). Of note, this distance has to be sufficient for the surgical users to comfortably operate. Current WDs from FGS systems range from ∼15 to ∼50  cm.^5^ Nevertheless, increasing the WD or FOV size can cause a loss of image sharpness unless zooming lenses are adapted. Important parameters such as the aperture size, f-number of the lens, and zooming capabilities can be adapted on this block through additional optical elements.^12^ Nevertheless, imaging lenses, especially those covering large FOVs, are prone to optical aberrations such as radial distortions.^12^ The presence of distortion is innate to imaging lenses, and when it is not corrected through the lens manufacturing process, it must be corrected through either post-processing algorithms and/or additional correction lenses.^39^^,^^40^ The selection of this lens block also has a great impact on the spatial resolution and sharpness of the detected features.^12^

Another parameter dictated by the optical arrangement of the detection block is the depth of field (DOF), which conveys the range of z distances from the focus plane at which image quality is preserved or lost. Similar to the human eye, detector blocks better resolve images that are in focus, but when objects are too far or too close, beyond the focus region, the resolution quality is lower.^41^ Nevertheless, optical elements can help correct for this loss in spatial resolution quality when focusing mechanisms are implemented into the detection block either manually or automatically. Of great importance here, the natural human instinct is to move closer to objects to better visually resolve them, but in the case of FGS systems, their particular lens system has an optimal WD and a finite DOF.^42^ Hence, placing the FGS detection system closer to the FOV (e.g., the patient) does not necessarily improve the spatial resolution. The image would tend to get brighter, but the image quality would degrade. This unintuitive behavior of most FGS systems requires some training and experience for appropriate interpretation by the user. It is imperative that users appreciate that the plane of focus defines the ideal WD for most systems and that the range of distances that are useful is known.

As illustrated in Fig. 2(b), components such as dichroic mirrors (D1 and D2), relay lenses (RL), and optical filters (F) are used in addition to the collection optics. In summary, dichroic mirrors (D1 and D2) and fluorescent filters isolate specific wavelengths to their respective detector paths, and their properties must be determined based on the emission profiles of the target fluorophores. Relay lenses then help to properly distribute these emissions into the detector’s (sensor) active area. In the example of Fig. 2(b), three detectors are used, one for color images and two for detecting different fluorescent wavelengths.^5^ Hence in this case, two different fluorescence contrasts with different wavelength emissions can be visualized as long as their excitation ranges are covered by the illumination block. Nevertheless, the use of a single fluorescent detector and a color detector is a more common arrangement. In an ideal scenario, filters should block everything except the target wavelengths, so filters that have high transmission (%T) and optical density (OD) are preferred as they can better isolate the weak signal coming from the fluorescently labelled tissue from the strong excitation light and the background. Low OD filters can result into excitation light leaking into the fluorescent detectors. Despite their angle dependency, interference filters are often preferred due to their high transmission and OD.^12^

Another important consideration is that room lights sources (e.g., tungsten, LEDs, or halogen lamps)^5^ are commonly broadband, which means that they have wavelength components that can overlap with the emission of the fluorophores. Unless FGS systems are equipped to isolate room light contributions, they should be operated under dark conditions for optimal performance or utilized with lights that do not overlap their known detection spectrum. This is specifically why most FGS systems are not able to operate in the presence of windows that let in sunlight or in settings with brighter tungsten lights.

The use of gated detectors in conjunction with pulsed-illumination sources or isolation through frequency modulation can aid in the removal of room light wavelengths.^5^^,^^36^^,^^43^ To quantify the contribution of visible room lights, a target that has a similar emission to the fluorophore of interest (e.g., ICG) but that is excited in the visible regime (e.g., 350 to 700 nm), in which common light sources typically operate, can be used. In systems in which detectors can be on while illumination sources are off, a background image can be taken with and without illumination to characterize filtering quality. FGS systems commonly opt for pixelated detectors such as CCDs and CMOS sensors. Important parameters to consider are the active area size, bit depth, sensitivity and linearity, readout rates, background noise, signal amplification, and time-gating capabilities.^12^ For a given sensor, the image exposure time and gain provide variation in how the detected fluorescence intensity is mapped into the digital pixel response. The fluorescence intensity levels are related to the fluorophore’s concentration and excitation light irradiance; hence by varying the concentration or irradiance, the output intensity levels can be varied. From a system perspective, the distance from the light source to the imaging plane changes the excitation irradiance, whereas the optics and sensor settings change the mapping of the resulting fluorescence to the pixel response. Understanding how the imaging system responds to various levels of fluorescence and their correlation to *in-vivo* fluorophore concentrations is key for application-specific performance assessments and cross-system comparisons. In this regard, sensitivity is related to the lowest level of fluorescent signal that can be detected by the FGS system. The data acquired by the image sensor is relayed to the processing block, which provides interpretation, display, and analysis of the detected fluorescence and overlay to the white light image.

#### 2.2.3 Processing blocks

Once data are collected by the detection block, each image has to be processed when appropriate and further displayed. FGS systems commonly display a white light image that represents tissue colors as seen by the naked eye, whereas fluorescent images are overlayed on white light images for display purposes and to better guide resection areas. This overlay process happens at the processing block. Images can also be adapted into different colormaps as exemplified in Fig. 2(c). Several important considerations for FGS systems are the sensor digitization process, readout post-processing, image compression, and use of look up tables (LUTs) employed for image display. The implementation of this imaging-to-display pipeline greatly alters the information relayed to the surgeon and is often completely opaque to the user. This pipeline often accounts for significant differentiation in systems and performance differences when comparing two systems or even for the same system in different operational modes. The compression of the fluorescence imaging data along with the implementation of commercially proprietary LUTs (e.g., reduction in bits used, bit-compression, and non-linear processing) each lead to limitations in the ability to even analyze information quantitatively in system comparisons due to the loss of data, saturation effects, and non-linear mapping of detected fluorescence intensity. For example, standard LUT implementations drastically change the reported signal to noise (SNR) and contrast to noise (CNR) metrics given common baselining and scaling of the camera sensor data for display purposes. Additionally, common LUTs display FGS signals with transparency levels below an arbitrary threshold, below which the signal is deemed to be unimportant background, so the user is unaware of anything below these levels. Although the use of image compression and LUTs is generally necessary for useful image display of the fluorescence emission, the translation of clinical studies into the standard of care could benefit from system manufacturer transparency of the imaging-to-display pipeline, for example, including the LUT as metadata for the image. Analysis of how standards can be used for LUT optimization and user interpretation is likely a full study in itself, beyond this work.

#### 2.2.4 User processing block

The user control block refers to the image display and feedback functions that allow for the control of detection, illumination, and image processing blocks. FGS systems commonly employ different LUTs, which can be assigned as different modes within the interface. Additionally, some systems have user controlled magnification or reference setting tools that alter the image display. The display and interface of the FGS system has significant impacts on the usability of the device, providing the direct communication of fluorescence contrast information at the clinical point-of-care. Understanding of how the applied tools influence contrast perceptions and surgical decision should be considered. As more quantitative FGS applications continue to be developed (for perfusion flow kinetics for surgery guidance), careful attention should be given to the user interface and information processing to ensure consistent use in surgical decision making.

### 2.3 Assessment of FGS System Parameters: Phantoms and Reference Targets

Fluorescence phantoms and reference targets are commonly used for FGS system performance assessments and comparisons. Over the past decade, the field of fluorescence phantoms has witnessed continuous development, with a transition from liquid and turbid phantoms to solid phantoms as the preferred reference for performance assessment.^44^ Liquid phantoms have traditionally relied on the use of lipid emulsions and blood to mimic tissue scattering and absorption. The primary drawback of liquid and turbid phantoms is their short shelf-life (hours to days), which leads to single-use functionality, being prone to human preparation error, and reproducibility issues. By contrast, solid phantoms based on polyurethanes, silicone, and plastics generally provide long-term stability (months to years).^11^ Recently, the use of cured two-part polyurethanes and 3D printed photocurable resins has enabled the manufacturing of long-term stable targets with tissue mimicking absorption and reduced scattering coefficients.^45^ To simulate the fluorescence emission of fluorescence contrast agents, both quantum dots and laser dyes have been used. Quantum dots provide the advantage of photostability with tunable emission wavelengths along with the drawbacks of broadband visible-NIR excitation and high costs.^9^ By contrast, laser dyes can mimic both the emission and excitation of the fluorescence contrast agents with the drawback of inherent photobleaching due to the nature of being organic dyes. An ideal dye might provide an avenue for balancing the drawbacks of both quantum dots and laser dyes, but no viable candidates have been identified in the literature for current FGS applications. To perform the tests suggested in the recent consensus report,^12^ evaluation of FGS systems, various tools, phantoms, and reference targets are needed. Figure 3 provides a summary of the qualitative parameters that are tested as part of the FGS system assessment. In brief, the assessment focuses on excitation illumination quantification (wavelength, power), image resolution, uniformity, and depth of field and contrast agent concentration sensitivity, linearity, and depth sensitivity.

The excitation spectra and FOV irradiance [Figs. 3(a) and 3(b)] are characterized using a spectrometer and optical power meter, respectively. FOV uniformity of the illumination-detection blocks can be initially verified through a flat and homogeneous fluorescent surface in vitro, as seen in Fig. 3(c), before moving to patient topography. Distortion testing can be performed with a grid of equidistant points or lines, as shown in Fig. 3(d). Image sharpness of imaging systems can be quantified through fluorescent spatial resolution targets (e.g., 1951 USAF target), such as the one displayed in Fig. 3(e), which contain varying spatial frequencies and size groups.^46^ To know the DOF of an FGS system, a similar fluorescent resolution target to the one in Fig. 3(e) can be used and measured at closer and further distances than the plane of focus or WD. As described in Fig. 3(f), in an ideal case, the image contrast would be the same across all distances; however this is not true for any imaging system, so knowing the “in focus” range is important for surgeons to better utilize FGS devices. For the characterization of sensitivity, a phantom similar to the one shown in Fig. 3(g), in which fluorophore concentrations are varied within media that simulates the optical properties of tissue, can be used. In addition to knowing which is the lowest intensity detectable by the system, understanding the depth at which a signal can be reliably detected is of importance. Even when the use of NIR and NIR II wavelengths helps to increase the imaging depth for optical imaging setups, optical arrangements have limited tissue penetration depth compared with other imaging modalities due to the turbid nature and optical properties of tissue. Therefore, understanding an FGS system imaging depth at a determined concentration is important and can be done through a phantom such as the one displayed in Fig. 3(h), where the depth variations are induced by increasing the depth of tissue-like material under which the fluorophore is embedded. Phantoms such as these are also appropriate for margin assessment and signal to background ratios, in which a well delineated and low background image in respect to the fluorophore regions is desired to produce a higher contrast. The use of these phantoms with a simplified geometry provides well-defined optical parameters for system assessments. Anthropomorphic phantoms that mimic *in-vivo* conditions could be further used to provide application-specific performance metrics for characterization and comparison.

For this study, the commercially available reference targets (QUEL Imaging, New Hampshire, United States) are made from 3D-printed material that incorporate fluorophores, scatterers, and absorbers, as described in Ref. 47. The main material is a fluorescent photocurable resin with tunable optical properties. To create the resins, first a base photopolymer resin is selected, and then pre-dispersed fluorophores, absorbers, and scatterers are incorporated. Resins designed for liquid crystal display (LCD), masked stereolithography (MSLA) and digital light processing 3D printers with a curing wavelength of 405 nm were used. For pre-dispersion, stock solutions of the additives were prepared in dimethyl sulfoxide. Stock solutions can vary in concentration and amount to define the optical properties of the 3DP resin. The 3D models to be printed are then designed for each phantom and printed through an MSLA printer that uses an inverted lithography arrangement with an LED matrix (405 nm) and LCD screen for curing. For 3D printing, the exposure times and layer height range from 2.0 to 15.0 s and 0.01 to 0.10 mm, respectively.

## 3 FGS System Assessment Methods and Results

**Fig. 4:**
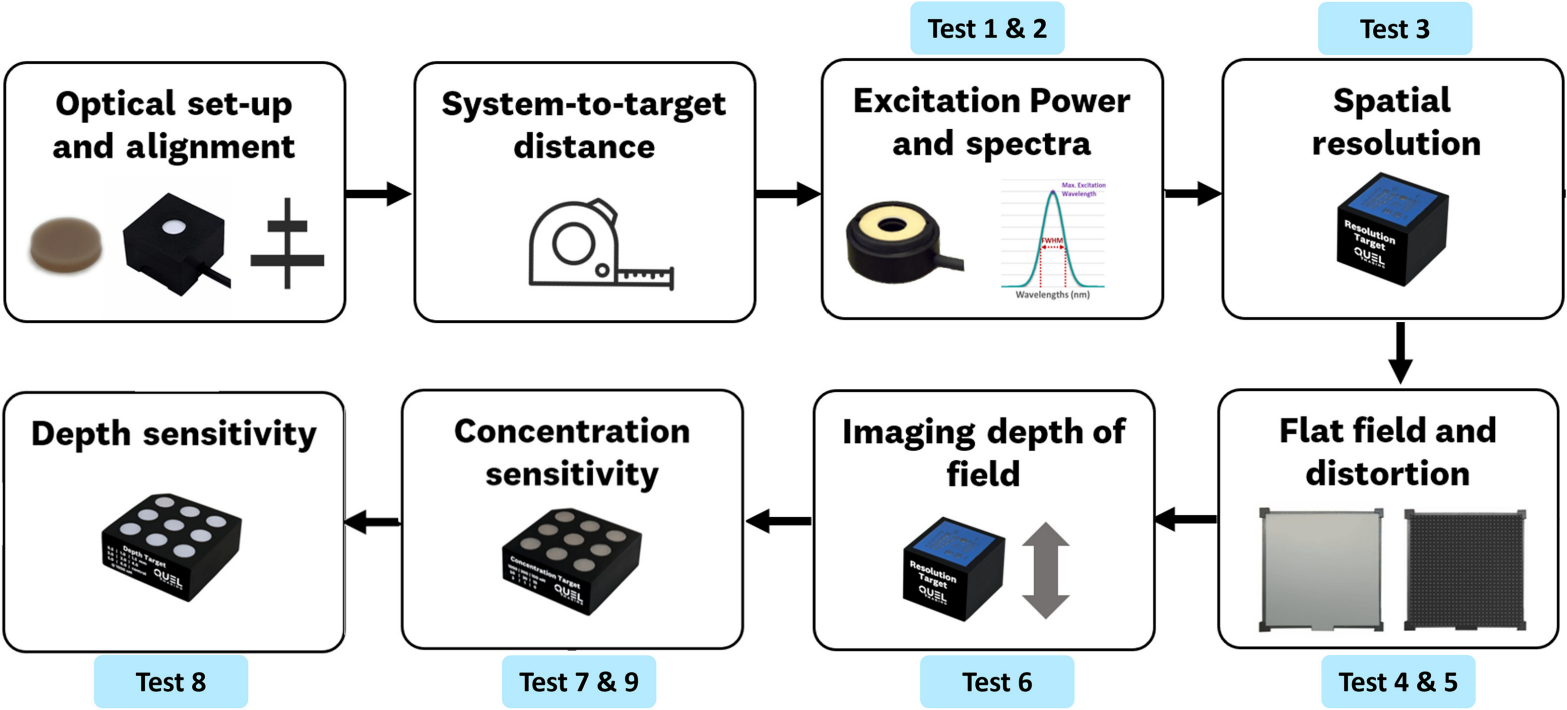
Visual flow diagram of the utilized testing protocol. The protocol provides assessment based on parameters suggested by a consensus report.^12^

The testing protocol and obtained results from the characterization of five different FDA approved systems are herein described. The used devices are Fluobeam (Fluoptics Imaging, France), EleVision (Medtronic, United States), OnLume Avata (OnLume Surgical, United States), SPY-PHI (Stryker, United States), and Fluobeam LX (Fluoptics Imaging, France). Throughout this manuscript, the FGS systems are referred to as system 1 (Fluobeam), system 2 (EleVision), system 3 (OnLume Avata), system 4 (SPYPHI), and system 5 (Fluobeam LX). Each system was characterized through nine main metrics: (1) excitation bands and (2) illumination power, (3) spatial resolution and sharpness, (4) uniformity, (5) spatial distortion, (6) optical DOF, (7) sensitivity and linearity, (8) imaging depth, and (9) signal to background ratio. Figure 4 provides a visual flow diagram for the testing protocol. For consistency, the selected modalities and parameters used for data acquisition per each system were kept the same across the different testing scenarios. The set parameters per each system are provided in Figs. S4 and S5 in the Supplementary Material. Additionally, procedure steps, which can be found in Fig. S5 in the Supplementary Material, were followed in the same order for each system. The different tests were quantified through *in vitro* ICG fluorescent phantoms prepared by QUEL imaging (White River Jct, Vermont, United States). The description of each phantom and the data acquisition procedure for each parameter are provided in the following subsections.

### 3.1 Tests 1 and 2 - Excitation Bands and Illumination Power Density

The spectral distribution of the excitation light was measured through a FLARE Miniature Spectrometer (Ocean Insight, Florida, United States) and fiber QP400-2-UV-BX (300 nm to 1.1  μm). Neutral density filter ND-05A (Thorlabs, New Jersey, United States) was used to avoid signal saturation. To avoid spectral bleed-through, a dark fabric was used to cover the illumination source head and setup.

**Fig. 5:**
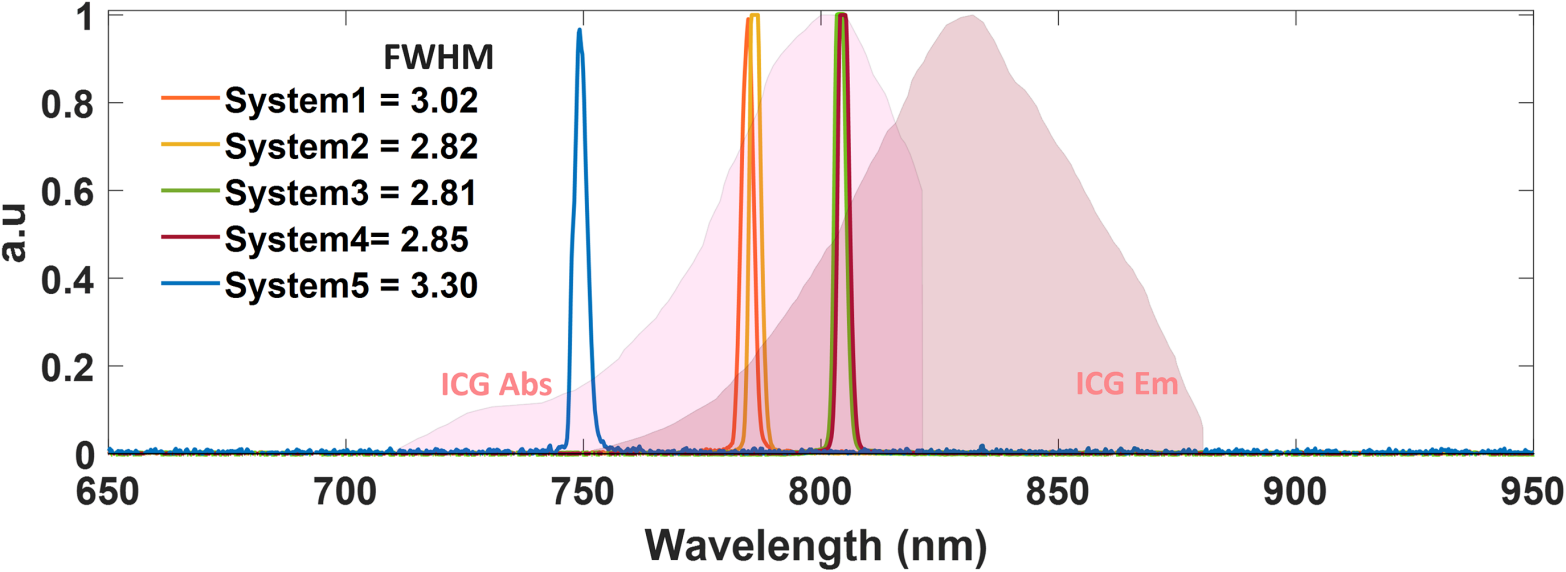
Spectral bandwidths of excitation sources for systems 1 to 5. These excitation spectra were measured per FGS system through a FLARE spectrophotometer device. FWHM values were calculated per system. Absorption and emission profiles of ICG are also displayed for reference. Spectra were normalized to their maximum value for display.

The acquired spectral distribution of the excitation sources is shown in Fig. 5(a) per each system. Of note, each of these systems has been cleared for ICG perfusion, as shown in Fig. S4 in the Supplementary Material, which gives the FDA clearance codes. The excitation and emission profiles of ICG were overlayed for visualization purposes. The FWHM of the spectral profiles, which is illustrated in Fig. 3(a) and values displayed in Fig. 5, were calculated in relation to wavelength units (nm). Results show FWHM values between 2.80 and 3.02 nm.

**Fig. 6:**
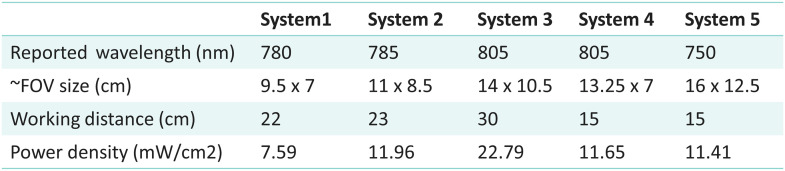
Summary of reported wavelength peaks and measured FOV sizes, used WD, and power densities for evaluation of FGS systems 1 to 5. Additional specifications are provided in the Supplementary Material.

Subsequently, optical power density was measured through power meter device 843-R (Newport, New Jersey, United States) and silicon photodetector 918D-SL-OD3R (Newport, New Jersey, United States). The calculated wavelength maximum was calibrated into the power meter, the photodetector was placed in the center of the FOV, and power was measured at the suggested WD. The estimated optical power densities of systems 1 to 5 together with important paramaters such as the estimated FOV size, manufacturer recommended WD, and reported excitation wavelengths are shown in Fig. 6.

### 3.2 Test 3 - Spatial Resolution and Sharpness

**Fig. 7:**
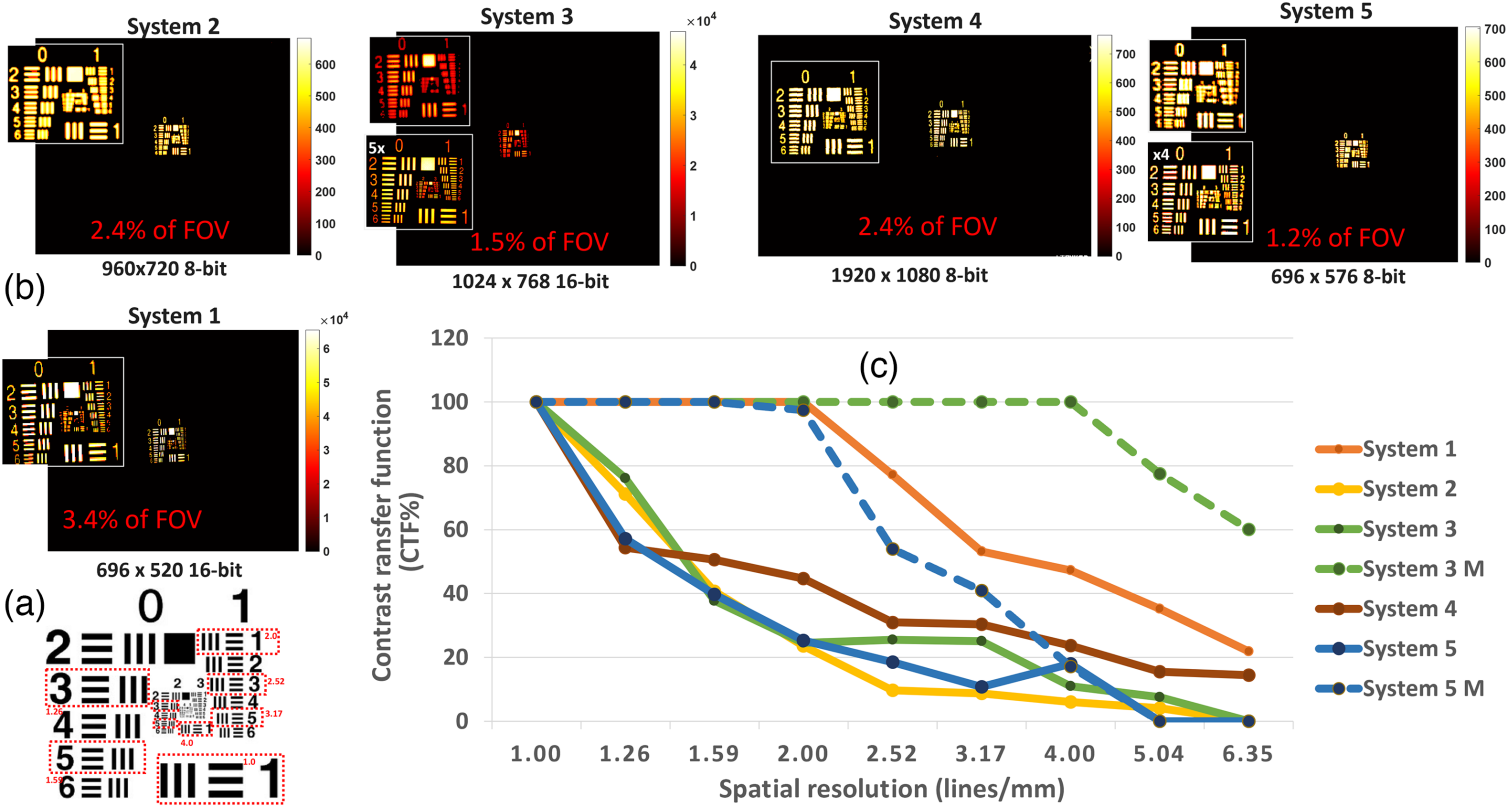
Spatial resolution test target (a) lp/mm used for quantification of spatial resolution and sharpness. (b) Images of resolution USAF 1951 fluorescent target as acquired with systems 1 to 5. (c) CTF results per selected group elements across the different systems. Systems 3 and 5 have high magnification (M) features, so results acquired with high M modes are also displayed for comparison purposes.

To characterize the spatial resolution range, an ICG based resolution target, Resolution USAF 1951 (QUEL Imaging LLC), was used [Fig. 3(e)]. As shown in Fig. 7(a), the target has the largest feature size at 1.0  lp/mm or 500  μm/line in group 0—element 1 and the smallest feature size at group 7—element 6 with 228.1  lp/mm or 2.19  μm/line.

Before using the USAF fluorescent target, the systems were placed in the “focus plane” at the above described WDs with the aid of a “coin” target [Fig. S1(a) in the Supplementary Material]. The target has a 2 cm thickness (z-plane), which is the same as the rest of the targets that were used throughout the protocol. Since focusing systems to the appropriate focal plane might take several minutes, an extra target is used to avoid photobleaching effects on the phantoms due to external light sources. To understand whether samples are photobleaching as they are used, a radiometric target from QUEL Imaging LLC, displayed in Fig. S1(b) in the Supplementary Material, is used as a control, and voltage values are recorded with each use of the phantoms. The phantoms contain ICG fluorescent mimicking material with matching ICG excitation and emission profiles.^45^ The material has an absorption coefficient ($\mu_{a}$) of $∼0.018\;{\mathrm{mm}}^{-1}$ and reduced scattering coefficient ($\mu_{s^{\prime }}$) of $∼1.4\;{\mathrm{mm}}^{-1}$ at 800 nm. The acquired images per each system are displayed in Fig. 7(b). For data acquisition and as summarized in Fig. S4 in the Supplementary Material, systems 1, 3, 4, and 5 allow for saving individual frames in “tiff,” “tif,” “jpg,” and “png” formats, respectively. For these systems, multiple individual frames were acquired per sample, and a single frame with a position of 1/totalframes was used for quantification. In the case of system 2, one of the acquired video frames (mp4 format) with position 1/totalframes was used. Systems 3 and 5 have magnification features, so an image using one of these magnifications was also displayed for comparison purposes. The respective bit depth and pixel resolution values were also displayed within the different images in Fig. 7(b). The features area of the target measures ∼1.5×1.5  cm, and the % of space occupied in relation to the full FOV is also estimated. To quantify the spatial resolution and sharpness, the elements with 1.0, 1.26, 1.59, 2.0, 2.52, 3.17, 4.0, 5.04, and 6.35  lp/mm were considered. These elements are also highlighted in Fig. 7(a). Per each acquired image, the background noise was thresholded with values between 0.1 and 0.4 of the maximum value. For each of them, a contrast transfer function (CTF %) value was calculated through Eq. (1), where r is the selected lp/mm region. Instead of considering a single maximum and minimum value, the min and max values represent the average of 20% of the highest and 20% of the lowest values in the defined lp/mm region, respectively.

$$\mathrm{CTF}(\%)=\frac{\left(\max(r)-\min(r)\right)}{\left(\max(r)+\min(r)\right)}*100.$$ (1)

For 1.0  lp/mm, the image sharpness quantified through CTF (%) was above 95% for all systems. Of note, this is at the recommended WD and plane of focus. As shown in Fig. 7(c), as the spatial resolution of the target increases, the contrast decreases toward the lowest resolution of 6.35  lp/mm. For systems 2 and 5, the CTF% decreases below 60% at 1.26  lp/mm, below 20% at 2.52  lp/mm and below 10% at the lowest resolution of 6.35  lp/mm. This was also true for system 3 without the use of the magnification feature. The highest variation between systems happens between 1.2 to 2.52  lp/mm. Of interest, systems that stayed with CTF(%) higher than 90% as the lp/mm increased were those that used magnification features and System 1 in which the sample was ∼3.4% of the FOV.

### 3.3 Test 4 - FOV Uniformity

**Fig. 8:**
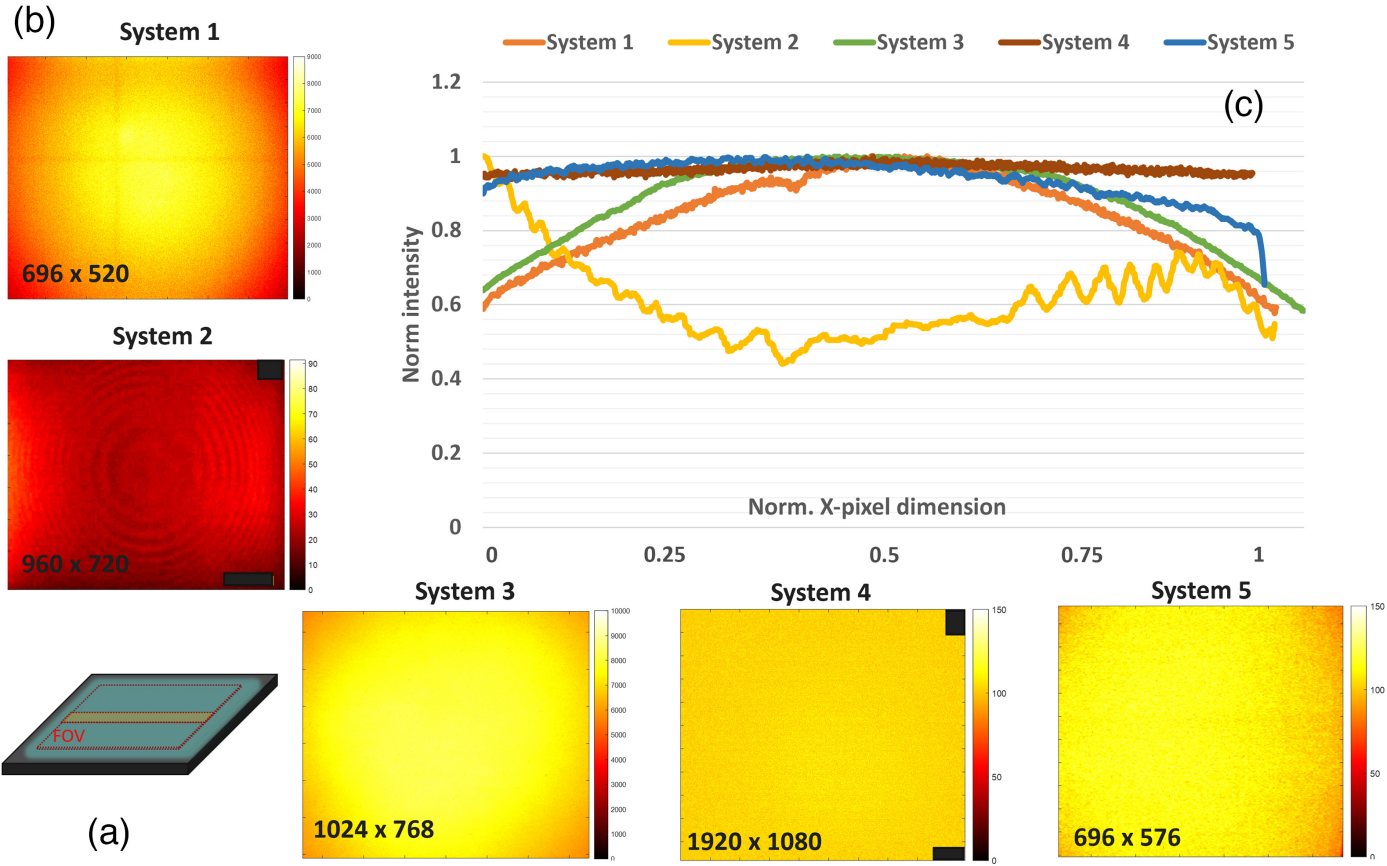
Depiction of homogeneity test phantom used (a) with dimensions $∼18\times 18\;{\mathrm{cm}}^{2}$, hence larger than the evaluated FOVs. (b) Acquired images of the homogeneous material with systems 1 to 5. (c) Plot of normalized intensity across the x pixel dimension of the sensor for each selected central region (shown in a) of the y axis. Pixels containing company logos were set to 0 for display here.

To calculate the uniformity of the FOV, a phantom with homogeneous distribution was placed at the WD of the systems. The phantom was of size ∼18×18  cm, which was larger than the targeted FOVs as depicted in Fig. 8(a). Images were acquired with parameters shown in Figs. S4 and S5 in the Supplementary Material per system. The used images are shown in Fig. 8(b). For each acquired image, the middle position of the Y pixel dimension was calculated. Then 50 pixels above and below were selected, and the mean was calculated per pixel position of the X axis. These values were then plotted as shown in Fig. 8(c) and used to estimate the homogeneity of the FOV. Since images have different x and y pixel dimensions, for display purposes, both the x and y axess were normalized. Results for system 4 show values between 0.95 and 1 for all pixel intensities, which means it shows a high level of homogeneity across the full FOV. System 5 displays intensities above 0.95 for values that fell between the first 75% of horizontal pixels and then values that decayed below 0.9 at the right-most border of the FOV. System 3 shows intensities that fell above 0.65 at the borders of the FOV and above 0.95 at the center of the FOV. A similar behavior was observed for system 1 but with lower intensity values at the left and right sides of the FOV. Finally, system 2 showed intensity values between 0.4 and 0.6 for ∼70% of the FOV with values above the 0.6 at the left and right borders of it.

### 3.4 Test 5 - Spatial Distortion

**Fig. 9:**
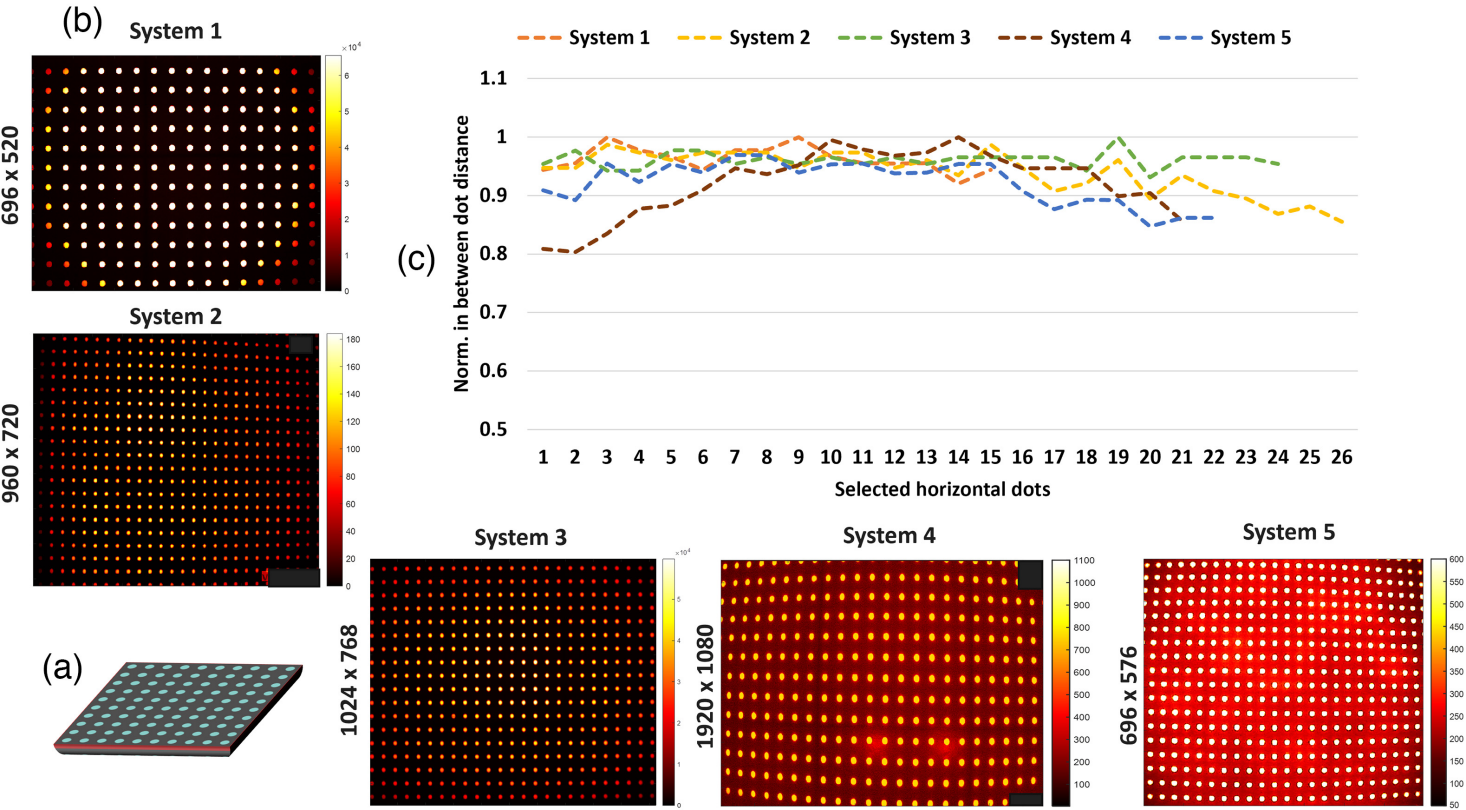
(a) Depiction of large area distortion phantom. (b) Images acquired by each system from the phantom with equidistant fluorescent points. The phantom was of dimensions $∼18\times 18\;{\mathrm{cm}}^{2}$, hence larger than the evaluated FOVs. (b) Acquired images with systems 1 to 5. (c) Plot of pixel distance between selected equidistant fluorescent points.

To test spatial distortion from systems 1 to 5, the homogeneous fluorescent phantom used in uniformity testing is covered with a black plastic sheet with equidistant fluorescent points. A depiction of the phantom is shown in Fig. 9(a). This way fluorescence only leaked from the dotted areas. The acquired images per each system are shown in Fig. 9(b). Once images were acquired, distortion estimation was done based on the average distance between neighboring dots across the horizontal FOV. This process is described in Fig. S1(c) in the Supplementary Material for a single system. Two lines of dots located across the middle position of the FOV y-axis were selected. The intensities of dots contained across the x-axis were plotted, with an example shown in Fig. S1(c) in the Supplementary Material. Then the position of each peak was estimated based on the local maximum and peak values. The pixel distance from one peak to the other was then estimated by subtracting their position. These pixel distances between positions were then plotted as shown in Fig. 9(c) and were used to estimate the level of distortion within the evaluated systems.

The resulting distances were normalized across the y-axis for visualization purposes, as shown in Fig. 9(c). In an ideal non-distorted image distances recorded between dots should be all equal to 1, which in the plot would be represented as a straight line across the selected horizontal dots. Results for system 3 show between dot distances above 0.9 for all dots, indicating that there was a lower level of distortion in the recorded image compared with other systems. This was also the case for system 1. System 2 displayed similar distance values above 0.9 for most dots, but values fell below this range on the right-most side of the FOV. System 5 shows distortion on the right side of the FOV as values fell between 0.85 and 0.9. Finally, system 4 displayed higher distortion in the left side of the FOV where values fell toward distances of 0.8.

### 3.5 Test 6 - Optical Depth of Field

**Fig. 10:**
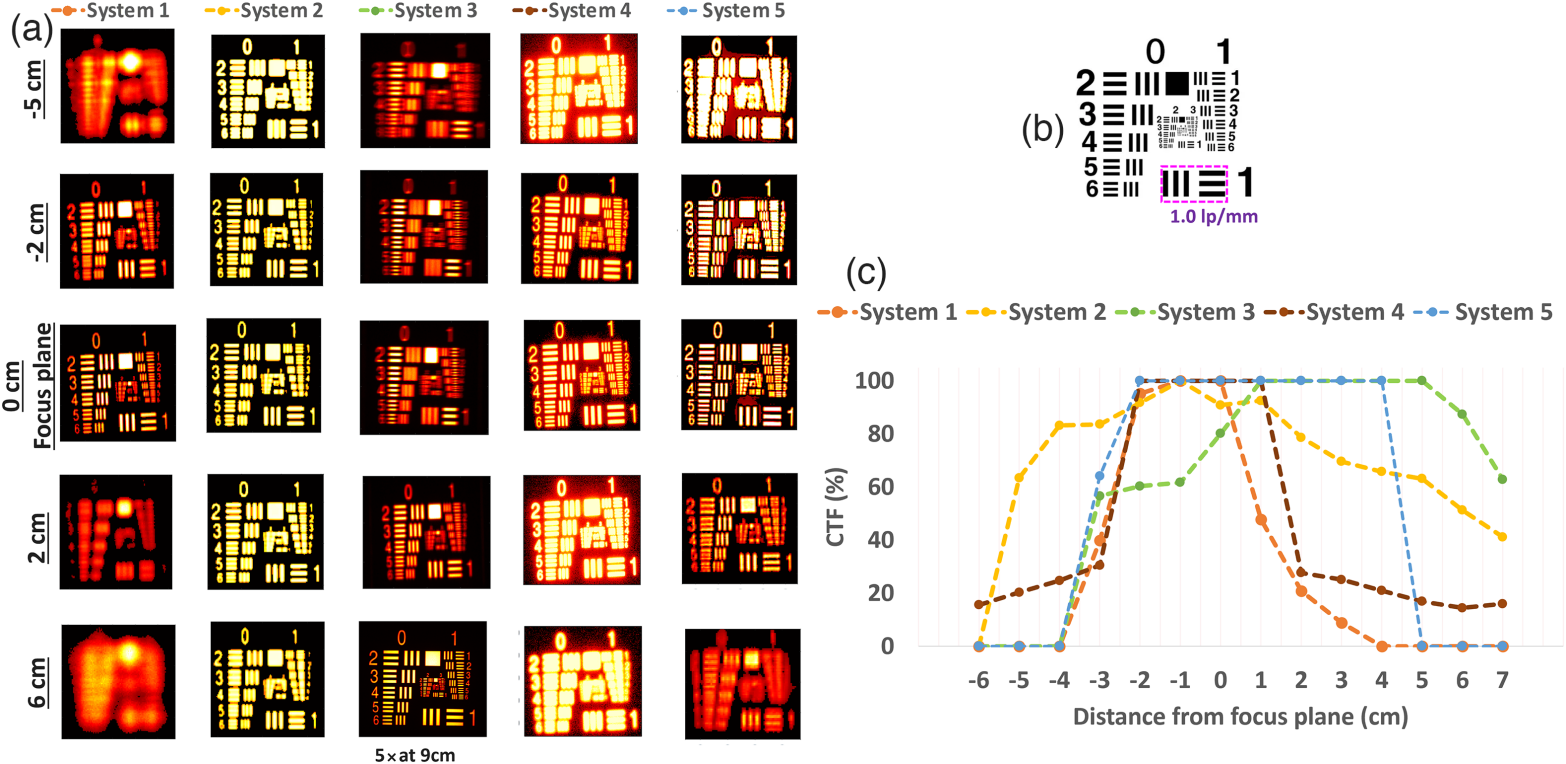
(a) Example of images acquired at −5, −2, 0 (plane of focus), 2 cm, and 6 cm. (b) Used elements for the quantification of the DOF metric. (c) CTF (%) as quantified for the selected 1.12  lp/mm resolution for all of the evaluated distances departing from the plane of focus at the 0 position. Images are cropped to the target site for better visualization.

To calculate the DOF, images were acquired for each system with the parameters mentioned in Figs. S4 and S5 in the Supplementary Material. In this case, the sample was the resolution target used in Fig. 7(a), but only the 1.0  lp/mm feature was of interest across the different positions, as depicted in Fig. 10. The systems were focused to their suggested WD, which corresponds to the values shown in Fig. 6. This plane of focus was defined as the distance 0. Then images of the resolution target were acquired at distances of 1 to 7 cm every 1 cm in both negative and positive directions. The negative values indicate distances further from the plane of focus but closer to the objective lens of each FGS system, and positive values indicate distances further away from the plane of focus and objective lens. Even though the evaluated systems had manual focusing arrangements for defined focal lengths, the sample was only focused when positioned at the 0-distance plane. This aims to illustrate the importance of considering the DOF in cases in which the surgeon focuses to a defined imaging z plane but either the patient movement or movement of the imaging head can change the distance in a negative or positive z position. This is of special importance for hand-held systems (e.g., systems 4 and 5). For those systems with magnification features (systems 3 and 5), magnification is not applied to cover the full FOV for all systems. CTF (%) values were calculated per each acquired image (at each z distance position) in a similar process, as described in test 3 with Eq. (1), but only for the 1.0  lp/mm element group 1. The CTF (%) values of both horizontal and vertical components were quantified separately, and the mean CTF (%) was calculated from them. Figure 10(a) displays example images acquired with each system at distances of −5, −2, 0, 2 and 6 cm, respectively. System 2 had a security feature indicating that the imaging head was too close to tissue; hence the considered negative distances go from 0 to −6  cm, and the positive distances go from 0 to 7 cm. The full set of images is shown in Fig. S3 in the Supplementary Material. Figure 10(c) shows the CTF (%) quantification results for all systems and mentioned z positions.

Systems 1 and 4 display higher CTF (%) for the 1.0  lp/mm element within a range of 0±2  cm. System 3 shows the highest CTF (%) when the distance between the imaging head and the sample increases from 1 to 5 cm from the set distance. System 2 shows the largest CTF (%) range, covering values above 60% from 0±5  cm, whereas system 5 performs the best at 1±5  cm.

### 3.6 Test 7 - Concentration Sensitivity

**Fig. 11:**
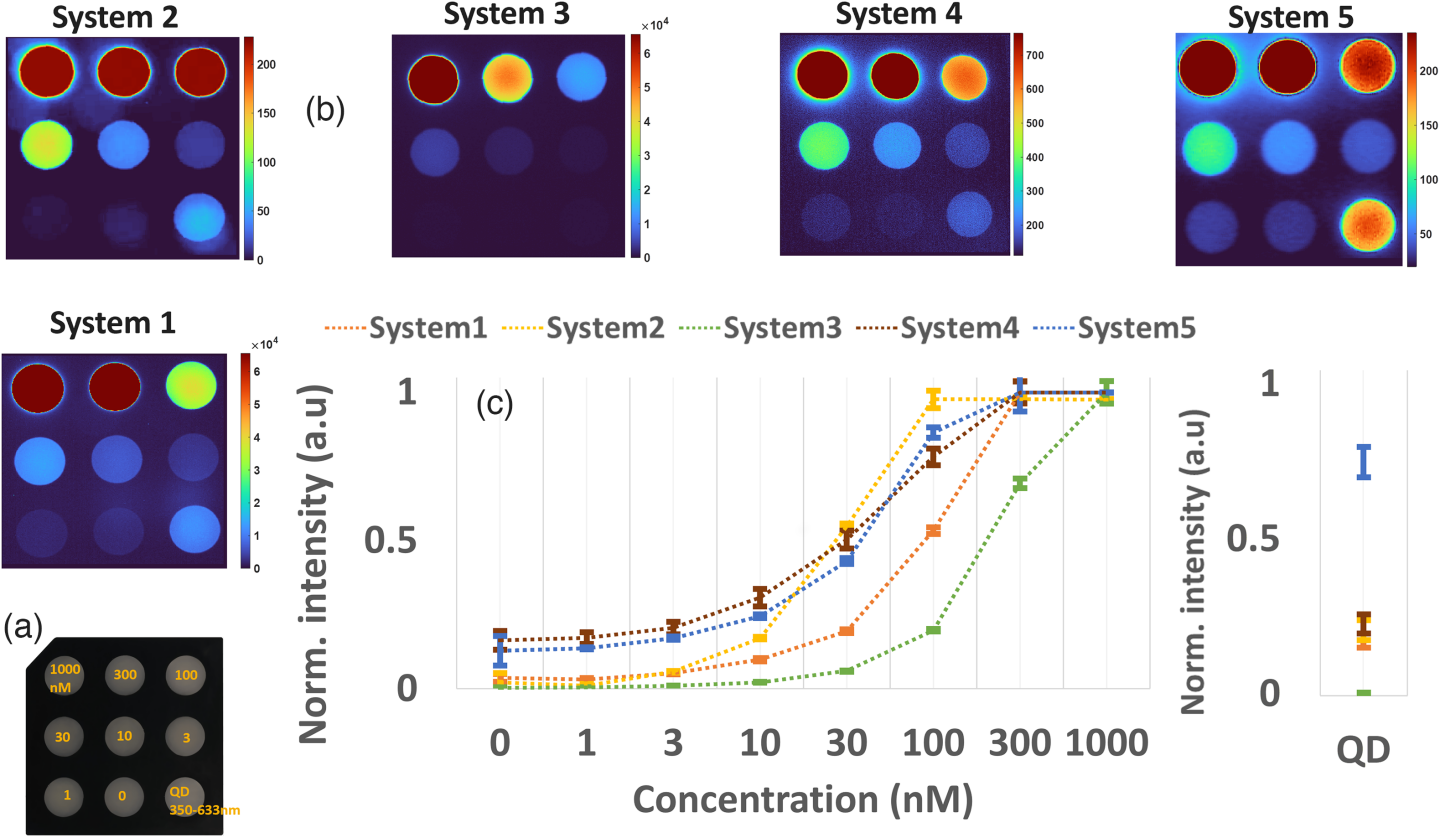
(a) Concentration variation imaging phantom with seven wells with different ICG nM concentrations, a 0 nm control well, and 800 nm emitting quantum dot well (QD). (b) Output images acquired from the phantom with each of the evaluated FGS systems. (c) Mean and standard deviation plot of each well per evaluated system. Calculated means and standard deviation per system were normalized to their maximum value for display purposes. The 800 nm quantum dots are a different probe than ICG with excitation from 350 to 800 nm; hence it is displayed separately.

For sensitivity testing, a varied concentration target (QUEL Imaging, White River Jct, Vermont, United States) was used. The target mimics ICG fluorescence in the presence of tissue optical properties with ($\mu_{a}$) of $∼0.010\;{\mathrm{mm}}^{-1}$ and reduced scattering coefficient ($\mu_{s^{\prime }}$) of $∼1.9\;{\mathrm{mm}}^{-1}$ at 800 nm. The phantom contains nine wells of 1 cm diameter, with decreasing concentrations of 1000, 300, 100, 30, 10, 3, and 1 nm. It also contains a control well with 0 nm and a well with 800 nm emitting quantum dots (QD800), which can be excitable across wavelength bands 350 to 800 nm. The sample was measured with each system using the same parameters and modes reported in Figs. S4 and S5 in the Supplementary Material. The sample is depicted in Fig. 11(a).

**Fig. 12:**
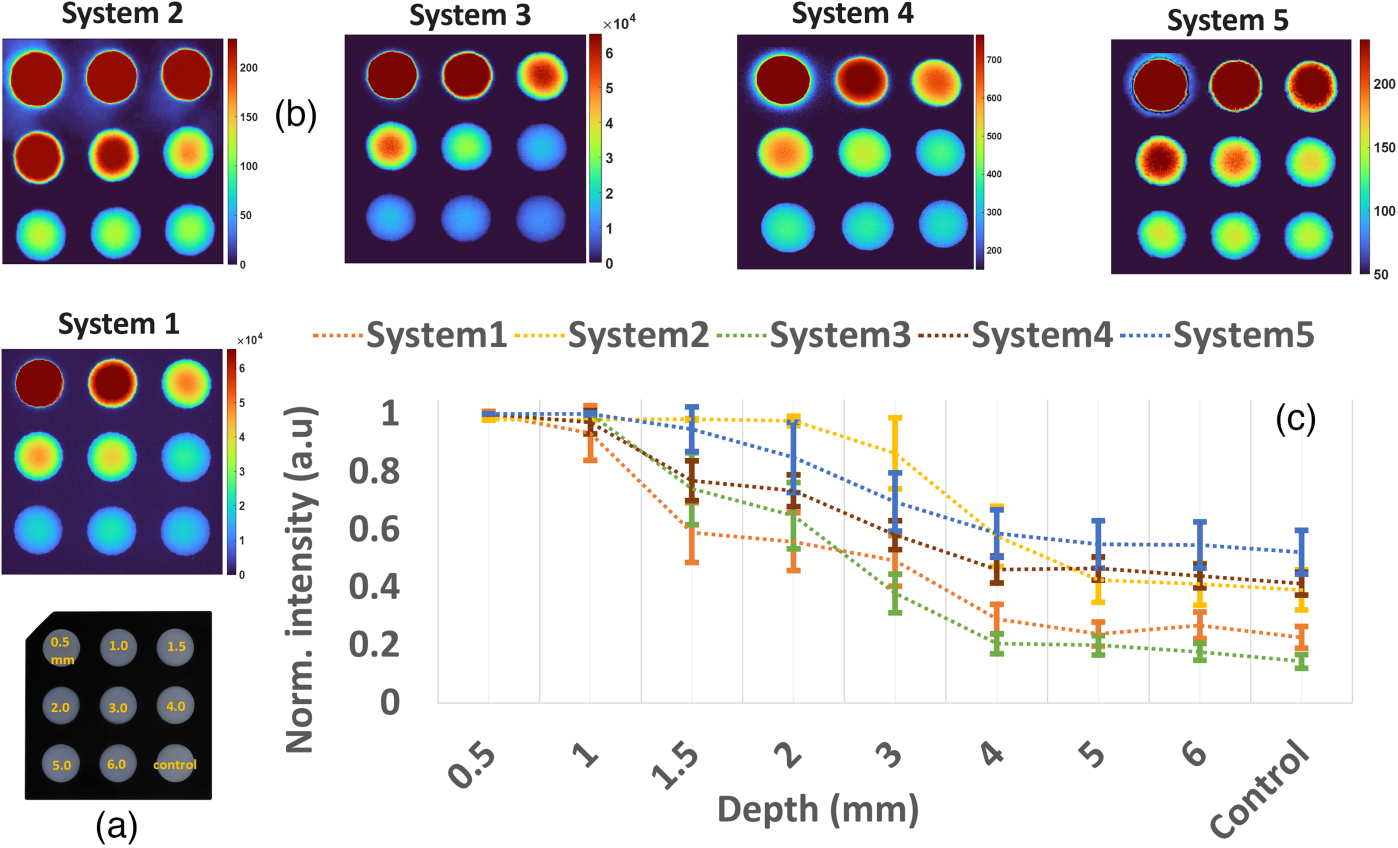
(a) Depth imaging phantom with eight wells with ICG 1000 nm at different depths and a 0 nm control well with 6 mm of mimicking material. (b) Images acquired from the phantom with each of the evaluated FGS systems. (c) Mean and standard deviation plot of each well per evaluated system.

The images of the concentration target, as acquired with each system, are shown in Fig. 11(b). In this case, the QD800 well is representative of the presence of room light sources that emit in the 350 to 633 nm range. Each system’s image is quantified by selecting well regions and calculating the mean intensity and standard deviation. Results for this quantification are plotted in Fig. 12(c) per each system. The mean and standard deviation values were normalized per system for display purposes. As shown in Fig. 12(c), eventhough systems 4 and 5 display higher sensitivity to the 1 nm concentration well, they display similar mean values for the 0 nm well. Systems 1, 2, and 3 display values approximating 0 for both 0 and 1 nm concentrations, which indicates a lower noise floor. In terms of saturation points, all systems display saturation at the 1000 and 300 nm wells. However for systems 2 and 3, the lowest concentrations of saturation were 100 and 30 nm, respectively. The importance of understanding the noise floor and saturation points is further discussed in the discussions and conclusions sections. The QD800 well that is excited from 350 to 633 nm showed higher intensity for system 5, indicating that, despite room lights being off, some light sources within these bands were present.

### 3.7 Test 8 - Imaging Depth

To approximate how deep each FGS system can image through tissue, a similar phantom to the concentration one was used, but it has nine different 1 cm diameter wells. In this case, each well contains two layers: a top layer of non-fluorescent tissue mimicking material (non-fluorescent resin) with thickness variations from 0.5 to 6 mm, having homogeneous $\mu_{a}=0.019\;{\mathrm{mm}}^{-1}$ at 800 nm, followed by a bottom layer with 1000 nm ICG-equivalent material with $\mu_{a}=0.021\;{\mathrm{mm}}^{-1}$ and $\mu_{s^{\prime }}=0.27\;{\mathrm{mm}}^{-1}$ at 800 nm. One negative control well containing no ICG and only 6 mm of mimicking material was used. This phantom is pictured in Fig. 12(a).

Acquisition parameters per each system are consistent with the ones previously used. Images of the phantom as acquired with each system are shown in Fig. 12(b). For quantification, the intensities of each 1 cm well were quantified, and the mean and standard deviation were estimated. The resulting values per well/depth are plotted per FGS system in Fig. 12(c). The mean and standard deviation values were normalized to the maximum value per system for display purposes. For FGS systems, the control well, which contains 0 nm ICG and only mimicking material, display values higher than 0 intensity counts, with systems 1 and 3 reporting the lowest level of signal at the control well. As the depth of ICG decreases from 6 to 0.5 mm, the mean intensity and standard deviation increase to 1 for all systems. Even though systems 2, 4, and 5 display higher intensity at the control well, they also show higher intensity at depths from 1 to 6 mm, with system 2 showing more intensity at the 2 and 3 mm depths. Further hypothesizing of why the control well was not showing ideal values close to 0 for all systems is addressed in Sec. 4.

### 3.8 Test 9 - Signal to Background Ratio

**Fig. 13:**
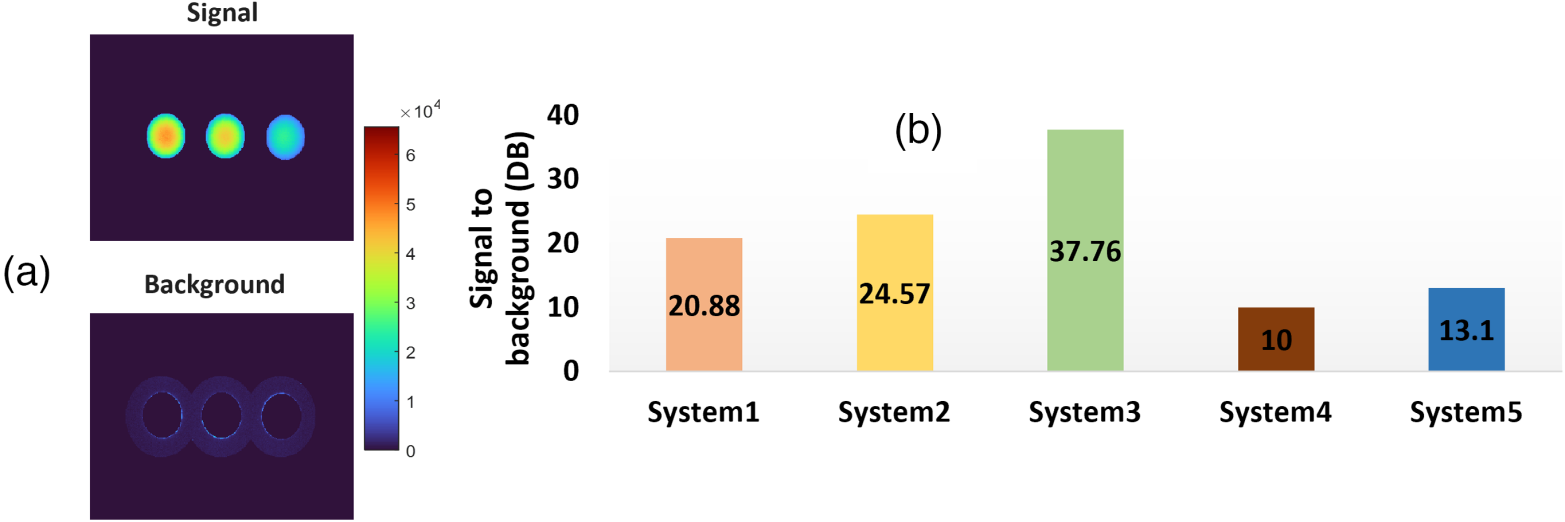
(a) Example of selected regions for signal (wells) and background (surrounding area). (b) Estimated signal to background ratios in dBs per each FGS system.

Even though test 7, in which concentration sensitivity was tested, gave an approximation of the noise floor of each FGS system, the images acquired from the depth phantom were also used for estimation of the signal to background ratio. For each image from an FGS system in Fig. 12(b), the wells containing ICG 1000 nm at depths of 2, 3, and 4 mm were used. A circular region with 0.5 units more than the well radius was selected for these 3 wells, as illustrated in Fig. 13(a). Then the well area was classified as the signal area and the surrounding regions as the background. Signal to background ratios (SBRs) of signal versus background are calculated by evaluating the ratio of the well intensity summed squared values over those of the background and displayed in dB units. Higher dB values are representative of lower background noise. Results for this test are displayed in Fig. 13(b) per each of the FGS systems.

Systems 1, 2, and 3 show signal to background ratios greater than 20 dB with system 3 displaying the lowest level of background. Systems 4 and 5 display the highest levels of background with 10 and 13.1 dB, respectively. An important factor was the use of LUTs, which computationally associate input values to color corrected outputs. The range defined in each LUT is important as it can aid in defining parameters such as the system saturation and noise floor. Figure S2 in the Supplementary Material shows, for an example FGS system, how the use of different LUT tables, which are often denoted in FGS systems as different “modes,” can have an effect on the saturation points and noise floor of the image.

## 4 Discussion

The tests completed in this study reveal differences between the system in their performance specifications and might even be used to trace back components of the system that need optimization. In tests 1 and 2 - excitation bands and illumination power density, the displayed minor variations in FWHM values, between 2.80 and 3.02 nm, suggest that this assessment may be of lesser impact. In addition to the measurement of the excitation spectrum of each FGS system, a more complete analysis could also describe the camera sensitivity to a wavelength range. This can be done through the use of a tunable light source or monochromator, hence allowing the user to understand or predict the performance for a different set of fluorophores. However, system FOV sizes did vary to a larger extend, from $9.5\times 7\;{\mathrm{cm}}^{2}$ to $16\times 12.5\;{\mathrm{cm}}^{2}$. Importantly, there is a clear increase in the minimum detected resolution when either zoom mode is used or there is a smaller FOV, as would be expected based upon the lens design. However, FOV and spatial resolution determination should correlate to the application planned and how much tissue region is needed to be seen in each video frame for the indicated surgical procedure. There is an inherent linkage between the spatial resolution, image sharpness, and DOF, and these tests were related to each other but revealed slightly different aspects. As shown through test 3—spatial resolution and sharpness, although a larger FOV can be beneficial for cases in which large areas are to be investigated, the use of zooming optics and/or smaller FOVs increases the minimum detectable resolution. From this study, features down to 1.59  lp/mm could be fully resolved by all systems with CTF (%) ≥ 40(%) when the sample accounted for 1.2% to 3.4% of the FOV, as shown in Fig. 7(c). This spatial resolution is expected to match the surgical need for imaging ICG perfusion within larger area tissues. To demonstrate how the z position of the imaging head can affect the resolution, test 6—optical DOF measurements were done with the fluorescent resolution target while changing z positions in reference to the focal plane. Systems with zoom capabilities (systems 3 and 5) showed a higher DOF at distances up to 6 cm away from the established WD, whereas systems 1 and 4 showed a narrower band of z distances at which the resolution for the 1.0  lp/mm feature was preserved. System 2 preserved the resolution for this feature across a broader range of distances.

This highlights the importance of FGS users understanding that, in terms of optical systems, a closer distance does not correlate directly to better resolution. Hence understanding the DOF of the FGS to be used is important for better imaging performance. This is especially critical for hand-held systems in which the lack of stability in a z position can cause a loss of resolution due to the DOF changes. Ideally manufacturers should contemplate the addition of ways to ensure that both hand-held and fixed imaging head options are automatically utilized at the appropriate height above the tissue or that the user is intrinsically guided to the right positioning. Another important aspect is the use of adaptive autofocusing capabilities. The systems tested here all had manual focusing that had to be adapted by the user depending on positioning. In this regard, the use of autofocus mechanisms similar to those found in most modern imaging cameras would be of benefit to avoiding DOF issues and spatial resolution loss. The intensity of the signal observed is related to (i) the optical power density (or irradiance) of the source across the FOV, (ii) the efficiency of the entire optical train that leads to the imaging sensor, and even (iii) the post processing and display LUT uses for the user. Not all of these can be independently assessed, but the relevant measures were successful. First, test 2 - optical power density showed that the evaluated systems ranged from 7.6 to $22.8\;\mathrm{mW}/{\mathrm{cm}}^{2}$ at WD ranging from 15 to 30 cm. This range of values matches a level that minimizes photobleaching but allows for imaging of most reasonable concentrations of ICG *in vivo*, as indicated by the injected dose. However, variations to the acquisition of the light signal and reception of the lens and optical train also modify how efficient each system is at detecting the emission. The fluorophore concentration sensitivity and imaging depth were evaluated as a way to integrate the entire optical efficiency of the system for how well it can detect levels of ICG. Test 7 - concentration sensitivity showed saturation and noise floor values for the evaluated systems. Of note, three of the five systems displayed values that matched expected outcomes for the 0 nM control well. Importantly, this test represents a negative control, hence indicating that values on this well might misrepresent the presence of low concentration ICG in areas where none is present. However, this can be corrected on the processing block through the use of LUT changes or color schemes that remove lower floor values that have no ICG signal. Hence understanding system noise floor or lower limit is key for better visualization of ICG stained tissue regions. In addition to the noise floor, saturation regions are an important parameter to understand. As shown in Fig. 11(c), the evaluated systems displayed saturation points above 100 nM ICG concentration for all systems. To accomplish better visualization of low concentration features, manufacturers often employ LUT tables to transform color-scales and further define saturation and noise floor points on the image. However, it is important that LUT table values (often referred as different modes within an FGS system) are defined not only through resolution targets but also through concentration and depth targets. Figure S2 in the Supplementary Material highlights how the implementation of LUT tables can change the perceived mean intensity and standard deviation values of both concentration and depth, as well as the saturation and noise floors of the image.

One of the more unique aspects of FGS as a modality is that the optical emission signal comes out from the tissue and the factors within the tissue such as blood volume can drastically affect the intensity detected. However, the design of systems can alter how the tissue attenuates the signal, and there are known differences in depth of sensitivity between systems. So assessment of the FGS signal depth measurements was revealed by test 8—imaging Depth, which provided insight into this factor for a standard “average” fixed value of tissue optical properties that varied in thickness over the ICG. For the evaluated systems and with the mentioned depth phantom, values could be reliably recovered at 4 mm depth. Values from 5 to 6 mm had values approximating the control well, which contained only media material and no ICG. For the control well, values were expected to be at 0 intensity; however, values between 0.2 and 0.3 were retrieved for systems 1 and 3 and between 0.4 and 0.6 for systems 2, 4, and 5. As shown in Fig. S2 in the Supplementary Material, this control value is something that could be addressed through the appropriate implementation of LUT tables that account for depth sensitivity. The interpretation of this background signal is complex, but it can inherently limit the lower level sensitivity of any of these systems. For all systems with the exception of system 3, measurements were performed with no room lights as recommended by users and as highlighted in Fig. S4 in the Supplementary Material. Even though low background light bleed-through is expected and is often corrected in the processing/image display blocks, it is important to reduce its effects. Figure 13(b) shows that the signal to background levels oscillate between 10 and 37 dB across the different systems for the depth target and non-saturated wells. An increase in background levels can reduce the contrast of features examined by the system; hence knowing background levels across different conditions can help to correct them in the processing block.

Finally, test 4 - FOV Uniformity and Test 5 - Spatial Distortion give insights into the importance of visualizing the uniformity of the FOV, as well as any distortions that might arise from the use of lenses. Figure 8 shows that most systems result in a Gaussian intensity distribution across recorded pixels, although system 2 displays a different distribution that might be due to use of Fresnel lens. Overall, the center position of the imaging FOV offers higher illumination homogeneity, which is key in exciting samples in a homogeneous manner, so fluorophore intensity fluctuations result from sample changes rather than irregularities on the excitation/illumination field. Lenses chosen for the detector path can also result in distortions of the detected FOV. Figure 9 shows that two of the five systems displayed radial distortion patterns. Even though distortion is mostly observed at the borders of the FOV, it is important to consider corrections within the detector path and/or disclose proper positioning of the sample of interest within the FOV. For this study, to guarantee that there were no significant variations caused by photo-bleaching effects of the samples, a radiometric target assessment was performed. An LED with a controlled voltage value was used to generate an intensity signal that matched the used phantoms, and these remained between 1.22 and 1.23 V from the beginning to end of the experiment, indicating sample photo-stability during the studies. To further guarantee this, system 2, which was the first system to be used, was used again at the end of the study, and the values remained stable. Nevertheless, further improvements of the used phantoms can be made. One improvement is the use of larger resolution features that can be assessed across the full FOV. This will provide insight into how the resolution changes due to illumination and/or distortion effects across the FOV. Though this can be accomplished by a separate test and moving the resolution sample to different locations of the FOV, it can result in tedious assessment for fast validation of systems. Another important improvement is the creation of a more convenient target for the DOF. Furthermore, the use of materials that replicate optical properties of tissue surface would also be beneficial, though this can prove challenging due to the varying optical properties of tissue at different regions and for different applications.

Finally, even though this study shows initial insight into a standardization procedure, further tests could be implemented for aspects not investigated here that might lead to better determination of individual component success/limitations within the systems. Another important future of implementation would be the use of pattern recognition algorithms that can quickly assess feature resolution as well as sample margins. These would be of benefit and will accelerate the data analysis turnaround and even could be built into the software of the systems for auto-calibration. For simplicity and fast turn-around time, this study used manual selection of regions and narrowed the scope to the use of specific features for some of the measured phantoms. For example, the DOF estimation was performed by monitoring only the 1.0 lp/mm elements due to the potential loss of resolution at higher lp/mm values. The use of this single region reduces potential variations that might arise from irregular manual region selection. Hence the use of pattern recognition algorithms could allow for the use of all features within the target for further analysis. This performance and standardization study was constrained to ICG based open surgery FGS systems and an additional system (system 5), in which the target contrast agent has overlapping excitation/emission bands to ICG. However, future work might involve similar testing across phantoms with different FDA approved contrast agents and systems that target their use. Another important aspect is that, for this study, specific imaging modes recommended by users were set for those systems with imaging mode selection (systems 2 and 3). However, when multiple modes are present, ideally manufacturers should evaluate each mode of the FGS system to determine changes on the different testing scenarios. This will likely become more important as the software features of systems are developed and customized by each manufacturer.

## 5 Conclusions

The importance of FGS system standardization has been highlighted throughout the tests completed in this work. This approach to standardization testing ideally might precede clinical trials and especially multicenter trials to allow for effective understanding of the limits to performance that exist for a single system and how different systems compare. Some of these processes for testing provide consistency in successful deployment for manufacturers and users. It is possible that these tests might work their way into manufacturers development pipelines through being part of their quality management processes and release testing and eventually provide objective evaluation data for regulatory agencies. The nine different features quantified here are each important for the system functionality, and all five systems tested here have performances that are suitable for their task. However, their use may also mitigate errors when changes in use between systems are made by surgeons. Each system is necessarily differentiated from the others as part of the development and sales pathway for commercial positioning, but these tests reveal key features of the systems that might be ideal for most to adopt. The optimal ones should be encouraged across the platforms for adoption; these include (i) clarity on magnification mode, (ii) homogeneity of illumination and detection across the FOV, (iii) intuitive user-directed warnings or guidance when the system is utilized outside the suitable tissue-device distance or when not in proper focus, and (iv) understanding of the display methods and how they affect user interpretation of the images. Further work on this topic is likely necessary in an iterative manner as the field of real-time use of FGS evolves and expands to further indications.

## Code, Data, and Materials Availability

Codes are available in a GitHub repository: https://github.com/ochoam2/StandardQuantifyFGS.git.
