# Supplementary material for: Assessment of open-field fluorescence guided surgery systems: implementing a standardized method for characterization and comparison
Source: J Biomed Opt. 2023 Sep 21;28(9):096007. doi: 10.1117/1.JBO.28.9.096007 (PMC10513724; doi:10.1117/1.JBO.28.9.096007)
Supplement: Supplementary file 1 [file JBO_028_096007_SD001.pdf]

Assessment of Open-field Fluorescence Guided Surgery Systems: Implementing a Standardized Method for Characterization and Comparison- Supplementary Material

Marien I. Ochoa[a], Alberto Ruiz[b], Ethan LaRochelle[b], Matthew Reed[a], Eren Berber[d], George Poultsides[e], Brian W. Pogue[a,*]

[a]University of Wisconsin Madison, Department of Medical Physics, 1111 Highland Ave, Madison, WI 53705
[b]QUEL Imaging, 85 N Main St, White River Junction, VT, 05001
[d]Cleveland Clinic - Marymount Hospital, 12300 McCracken Rd, Garfield Heights, OH 44125
[e]Stanford Medicine, Department of Surgery, 300 Pasteur Drive, Stanford, CA 94305

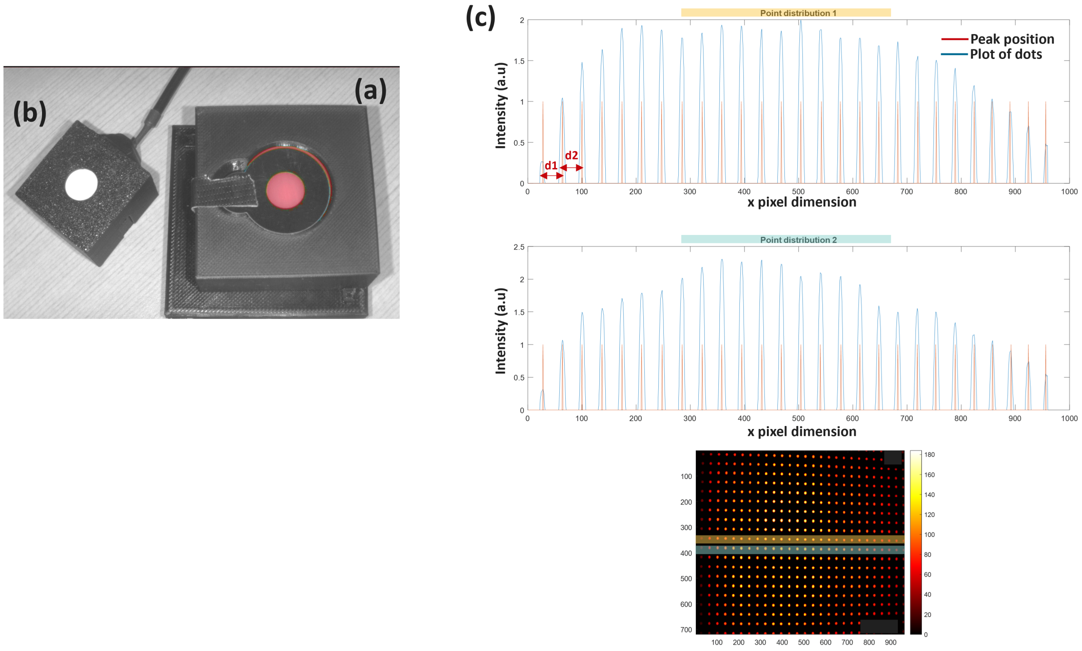

Fig 1 (a) Circular fluorescent intensity calibration target used for initial alignment of FGS systems.(b) Radiometric target used as control for potential photobleaching effects in ICG phantoms. (c) Illustration of quantification process for distortion target.

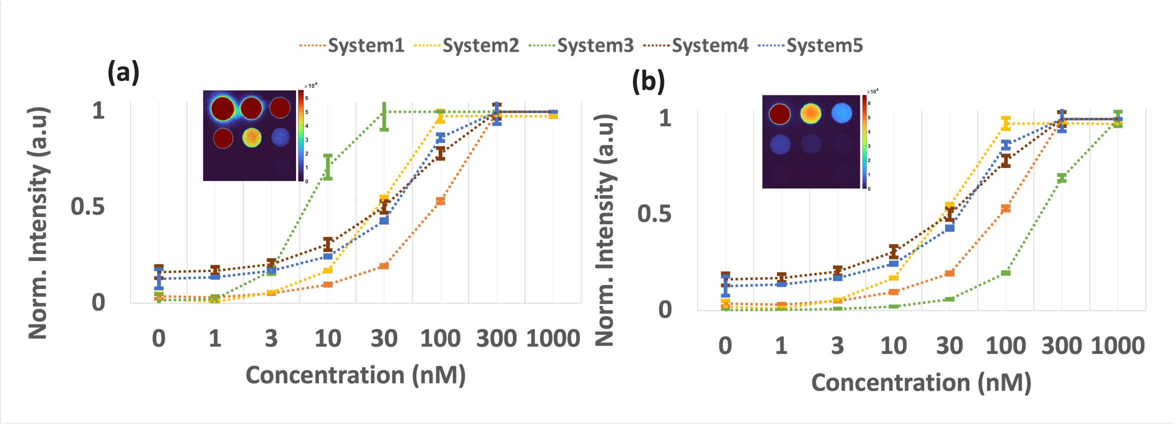

Fig 2 (a) Quantification of concentration phantom by System 3 when using "Leveled" mode. (b) Quantification of concentration phantom by System 3 when using "Direct" mode. Same phantom used but (a) involves use of LUT corrections. Leveled mode involves the use of LUT defined by the manufacturer while Direct is the output from the camera sensor without any LUT corrections. All other systems displayed for reference.

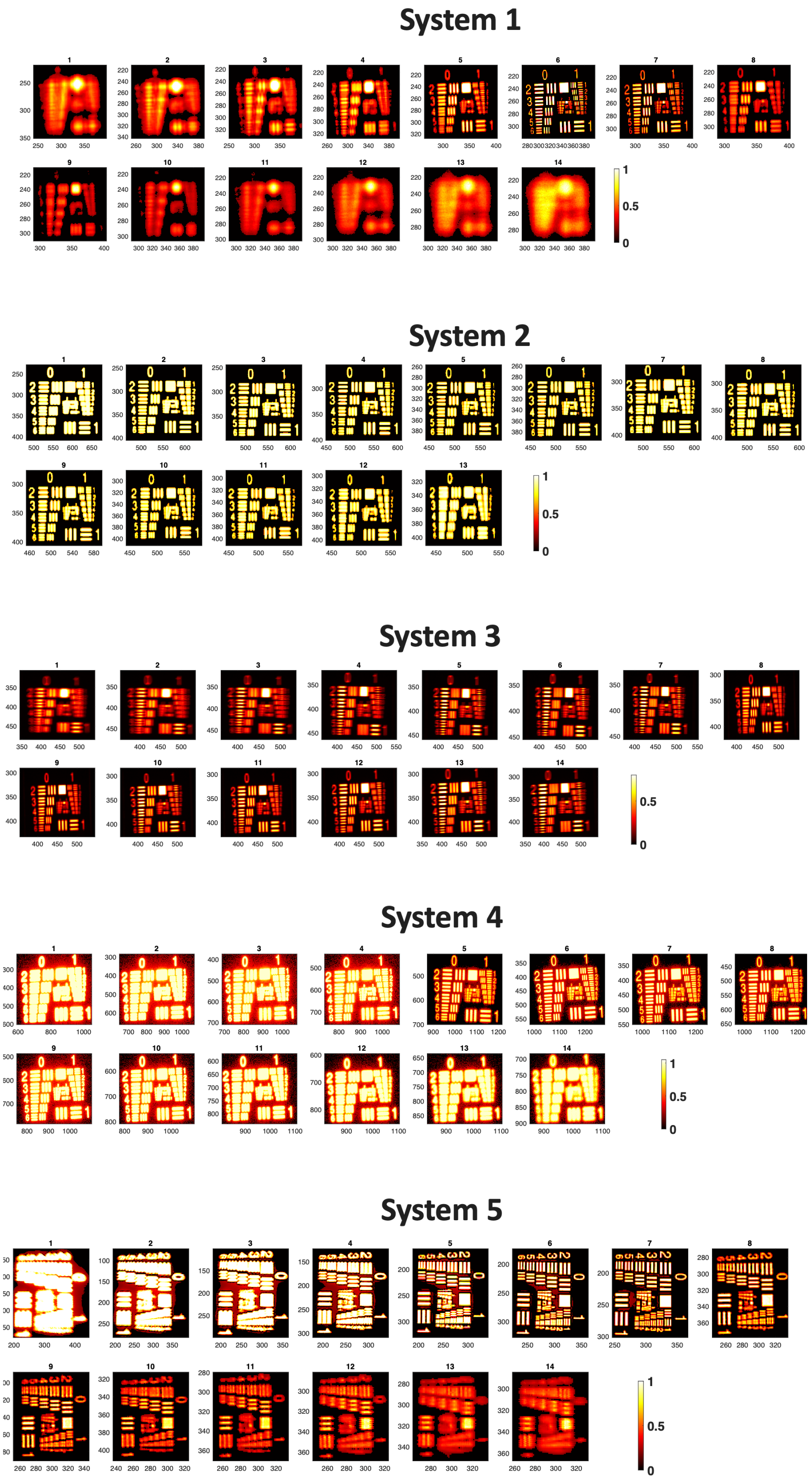

Fig 3 Full set of images acquired by each system for quantification of depth of field.

Comparison of technical specifications

		Fluobeam 800	EleVision IR	OnLume Avata	SPY PHI	Fluobeam LX
**System Name**		**Fluobeam 800**	**EleVision IR**	**OnLume Avata**	**SPY PHI**	**Fluobeam LX**
**FAD Approval year**		Preclinical	2019	2018	2020	2019
**Company**		Fluoptics	Iridium/Medtronic	OnLume Surgical	Stryker	Fluoptics
**Year of Purchase**		2011	2021	2020	2020	2019
**Illumination**	**Light source**	Laser	Laser	Laser	Laser	Laser
	**illumination wavelengths (nm)**	780nm	785	805	805	750
	**Maximum reported power output**	6.4mW/cm2	3000mW at 785nm	not disclosed	not disclosed	not disclosed
	**Optical Irradiance with ambient light (mW/cm2)**	7.59mW/cm2	14.96 mW/cm2	22.79 mW/cm2	11.65 mW/cm2 light is not off, dims	mW/cm2
	**Optical Irradiance without ambient light (mW/cm2)**	7.56mW/cm2	13.78 mW/cm2	22.36 mW/cm2	11.45 mW/cm2	mW/cm2
**Detection**	**Main Camera Model**	not disclosed	VS3 Iridium	not disclosed	not disclosed	not disclosed
	**Sensor Type**	CCD	CCD	CCD	not disclosed	not disclosed
	**Sensor Size**	not disclosed	not disclosed	not disclosed	not disclosed	not disclosed
	**FDA Subject Device #**	K132475	K191851	K192761	K202244	K190891
	**Target FDA approved contrast**	ICG	ICG	ICG	ICG	Parathyroid Autofluorescence
	**Maximum QE at target emission**	not disclosed	not disclosed	not disclosed	not disclosed	not disclosed
	**Camera read-out bits**	not disclosed	not disclosed	not disclosed	not disclosed	not disclosed
	**Fluorescent Channels**	1	1	1	1	1
	**Simultaneous Multispectral**	No	No	No	No	No
	**Variable Gain**	No (Auto scale)	Yes	not disclosed	not disclosed	not disclosed
	**Exposure Time**	80ms for measurement	not disclosed/automatic	not disclosed/automatic	not disclosed/automatic	50%
	**Operating Mode**	CW	CW	CW	CW	CW
**Optical Arrangement**	**Variable FOV - Zooming**	No	No	Yes	No	Yes
	**Focus mecanism automatic or manual**	Manual	Manual	Manual	Manual	Manual
	**Recommended Working distance to sample (cm)**	22cm	17 to 50cm	20 to 45 cm	15 cm	15 cm
	**Detection filter interchangeable**	No	No	No	No	No
**Output Video/Image**	**Pixel dimensions**	696 x 520 pixels	960 x 720 pixels	1024 x 768	1920 x 1080	696 x 576
	**Bit depth (bit)**	16	12 and 8	16	16 and 8	8
	**Real time white light and fluorescence overlay**	No	Yes	Yes	Yes	Yes
	**Recorded video data rate**	not disclosed	31.76 Mbit/s	not disclosed	7.74 Mbit/s	974.92 kbit/s
	**Video frame rate (Encoded FPS)**	~28 fps (~7 fps for 100ms exp)	22 fps	15 to 30 fps	30 fps	25 fps
	**Option to save video session**	Yes	Yes	Yes	Yes	Yes
	**Individual frame as image file option**	TIFF	H.264 MP4	TIFF / PNG	JPEG	PNG
	**Screen shot option**	Through Software / Windows	Through Software	Through Software	Through Software	Through Software / Windows
	**Binning options availiable**	No	No	No	No	No
**Ease of use features**	**Room light level**	On	Off	On	Off	Off
	**Background correction**	Option for processed data	Linear data processing	not disclosed	not disclosed	not disclosed
	**Assist Light**	Yes	Yes	Yes	Yes	Yes
	**Raw data export**	Yes	No	Yes	No	No
	**Number of display monitors**	1	up to 2	1	1	1
	**Size**	Handheld	~5 ft height/0.5 ft width	~5 ft height/0.5 ft width	Handheld	Handheld
	**Mount type**	Handheld	Mechanical Arm	Mechanical Arm	Handheld	Handheld
	**List of modifiable parameters with use**	Exposure, turn laser on, ambient assist on/off, monitor addition, record, save individual image, raw or processed data, autoscale, define directory, image name, display of previously acquired images, move next and previous image, eliminate image, Image processing tab.	Settings menu, swap images, turn laser on, flip image, imaging profile, view recordings, ambient assist on or off	Fluorescence mode on and off, video recording, individual image recording, acquisition time , zooming range, raw or processed data, data save, eliminate image, move between acquired images	Fluorescence mode on and off, video recording, individual image recording, manual focusing range, acquired images, ambient light/on off	Fluorescence mode on and off, video recording, individual image recording, exposure time, zooming range, additional display
	**On screen instructions**	No	No	No	No	No
	**USB data transfer port**	USB 2.0	USB 2.0	USB 3.0	not disclosed	not disclosed
	**Additional Imaging Modality**	N/A	Endoscopy	N/A	N/A	N/A
**Cost**		not disclosed	not disclosed	not disclosed	not disclosed	not disclosed

Fig 4 Table of specifications for the quantified systems.

	System Characterization Procedure Steps	Fluobeam 800		EleVisionIR		OnLume		SPY PHI		Fluobeam LX	
1	Put gloves on										
2	Write mode used for the system measurement	Not Defined		Plastic Surgery 785		Leveled/ Direct		SPY		N/A	
3	Align coin target/mask and focus to appropiate distance										
4	Distance from excitation to top of target	22	cm	23	cm	30	cm	15	cm	15	cm
5	Place radiometric target in FOV with coin target										
	* connect to and turn on supply										
	* voltage setting is 1.22-1.34V										
	* current limit should be 0.030A										
6	Set the power supply voltage so that the intensity	1.228-1.2230	V	1.258	V	1.239	V	1.24	V	1.221	V
	of radiometric target is slightly below coin target	100ms exp time									
7	Place Resolution Target leave radiometric										
	* select appropiate exposure time if applicable	80	ms	N/A	ms	5	ms	N/A	ms	50%	ms
	* adjust focus for all incoming measurements					505microse for grid					
	* record image										
8	Place Concentration Target										
	* Lower radiometric target by 0.01V.	1.236-1.238	V	1.248	V	1.239	V	1.241	V	1.221	V
	* 1000nM intensity > radiometric target										
9	Place Depth Target										
	* Lower radiometric target by 0.01V if necessary	1.236-1.238	V	1.23	V	1.238	V	1.241	V	1.22	V
	* highest intensity well > radiometric target			`							
10	Remove radiometric target from FOV										
11	Place Flatfield Target with black, then gray, then										
	diffusive sheets										
	* Align target in color camera if avaliable										
	* Acquire image with ambient assist ON										
	* Acquire image with ambient assist OFF										
	* Turn ambient asist ON if applicable										
12	Remove backgrounds and place dot matrix										
13	Remove dot matrix and place transparency grid										
14	Place Depth of Field spacers										
	* align coin target to initial wording distance 0										
	using height control block.										
	* measure for 0										
	* measure for +1 to +7										
	* measure for -1 to -7										
15	Measure spectral output										
	* Place dark fabric around										
	* Connect spectrophotometer and PC										
	* Connect optic fiber							*mW in on together			
	* Place fiber holder and fix fiber							due to dimming			
16	Measure excitation power							*white light lowers			
	* Leave dark fabric around	19.29	mW on	38	mW on	57.9	mW on	29.2	mW on	28.6	mW on
	* Set wavelength on Power Meter	19.22	mW off	35	mW off	56.8	mW off	29.6	mW on	29	mW on
	* Zero Power Meter when covered										
	* Excitation to cover Power Meter 2cm diam	7.594488189	d on	14.96062992	d on	22.79527559	d on	11.49606299	d on	11.25984	d off
	* Measure when ambient assist ON/OFF if applicable	7.566929134	d off	13.77952756	d off	22.36220472	d off	11.65354331	d on	11.41732	d on
				*PM diam (cm)	1.5						
					2.54 cm2						

Fig 5 Outline of utilized procedure for the study. Radiometric target values are displayed for each system. System 2 served as a control and at the end of the study radiometric target values in respect to phantoms were re-measured with this system and showed no significant change.
